# Supplementary material for: Corticothalamic neurons in motor cortex have a permissive role in motor execution
Source: Nat Commun. 2025 May 21;16:4735. doi: 10.1038/s41467-025-59954-1 (PMC12095750; doi:10.1038/s41467-025-59954-1)
Supplement: Supplementary file 1 — Supplementary Information [file 41467_2025_59954_MOESM1_ESM.pdf]

Supplementary Information

Corticothalamic Neurons in Motor Cortex Have a Permissive Role in Motor Execution

Figure S1

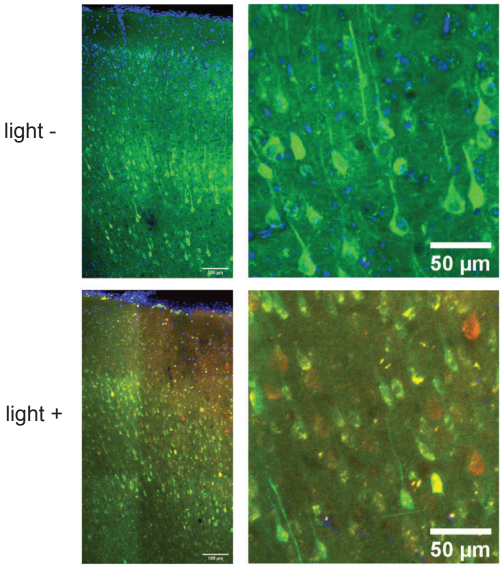

Figure S1. CaMPARI expression in M1 neurons

Representative images of CaMPARI levels in mice performing the wheel turning task in the absence (top) and presence (bottom) of photoconversion light. Green, baseline; red, photoconverted state. Right panels are zoom insets of left.

Figure S2

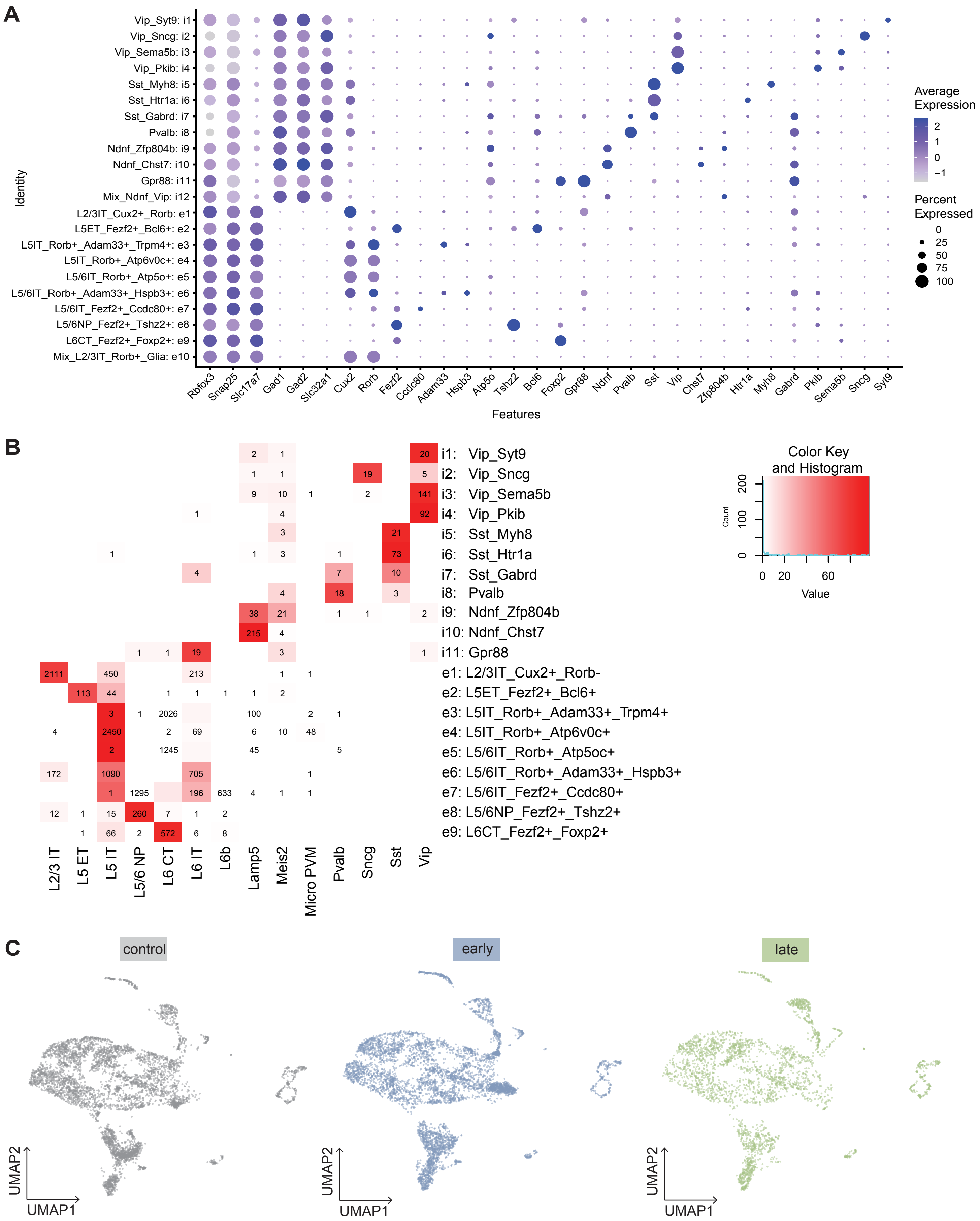
A
Average Expression
Percent Expressed
Identity
Features
Vip_Syt9: i1
Vip_Sncg: i2
Vip_Sema5b: i3
Vip_Pkib: i4
Sst_Myh8: i5
Sst_Htr1a: i6
Sst_Gabrd: i7
Pvalb: i8
Ndnf_Zfp804b: i9
Ndnf_Chst7: i10
Gpr88: i11
Mix_Ndnf_Vip: i12
L2/3IT_Cux2+_Rorb: e1
L5ET_Fezf2+_Bcl6+: e2
L5IT_Rorb+_Adam33+_Trpm4+: e3
L5IT_Rorb+_Atp6v0c+: e4
L5/6IT_Rorb+_Atp5o+: e5
L5/6IT_Rorb+_Adam33+_Hspb3+: e6
L5/6IT_Fezf2+_Ccdc80+: e7
L5/6NP_Fezf2+_Tshz2+: e8
L6CT_Fezf2+_Foxp2+: e9
Mix_L2/3IT_Rorb+_Glia: e10
Rbfox3 Snap25 Slc17a7 Gad1 Gad2 Slc32a1 Cux2 Rorb Fezf2 Ccdc80 Adam33 Hspb3 Atp5o Tshz2 Bcl6 Foxp2 Gpr88 Ndnf Pvalb Sst Vip Chst7 Zfp804b Htr1a Myh8 Gabrd Pkib Sema5b Sncg Syt9
B
Color Key and Histogram
Count
Value
i1: Vip_Syt9
i2: Vip_Sncg
i3: Vip_Sema5b
i4: Vip_Pkib
i5: Sst_Myh8
i6: Sst_Htr1a
i7: Sst_Gabrd
i8: Pvalb
i9: Ndnf_Zfp804b
i10: Ndnf_Chst7
i11: Gpr88
e1: L2/3IT_Cux2+_Rorb-
e2: L5ET_Fezf2+_Bcl6+
e3: L5IT_Rorb+_Adam33+_Trpm4+
e4: L5IT_Rorb+_Atp6v0c+
e5: L5/6IT_Rorb+_Atp5oc+
e6: L5/6IT_Rorb+_Adam33+_Hspb3+
e7: L5/6IT_Fezf2+_Ccdc80+
e8: L5/6NP_Fezf2+_Tshz2+
e9: L6CT_Fezf2+_Foxp2+
L2/3 IT L5 ET L5 IT L5/6 NP L6 CT L6 IT L6b Lamp5 Meis2 Micro PVM Pvalb Sncg Sst Vip
C
control
early
late
UMAP1
UMAP2

Figure S2. Gene expression and mapping of single cell RNA sequencing

A Average, purple gradient, and percent expression, size of dot, of marker genes in each cluster. **B** Number of cells mapped from clusters identified in aggregate sc-RNAseq runs (x axis) to clusters of the M1 cell type atlas (y axis). **C** UMAPs of scRNA-seq runs from each condition mapped to M1 cell type atlas; gray, control; blue, early training; green, late training.

Figure S3

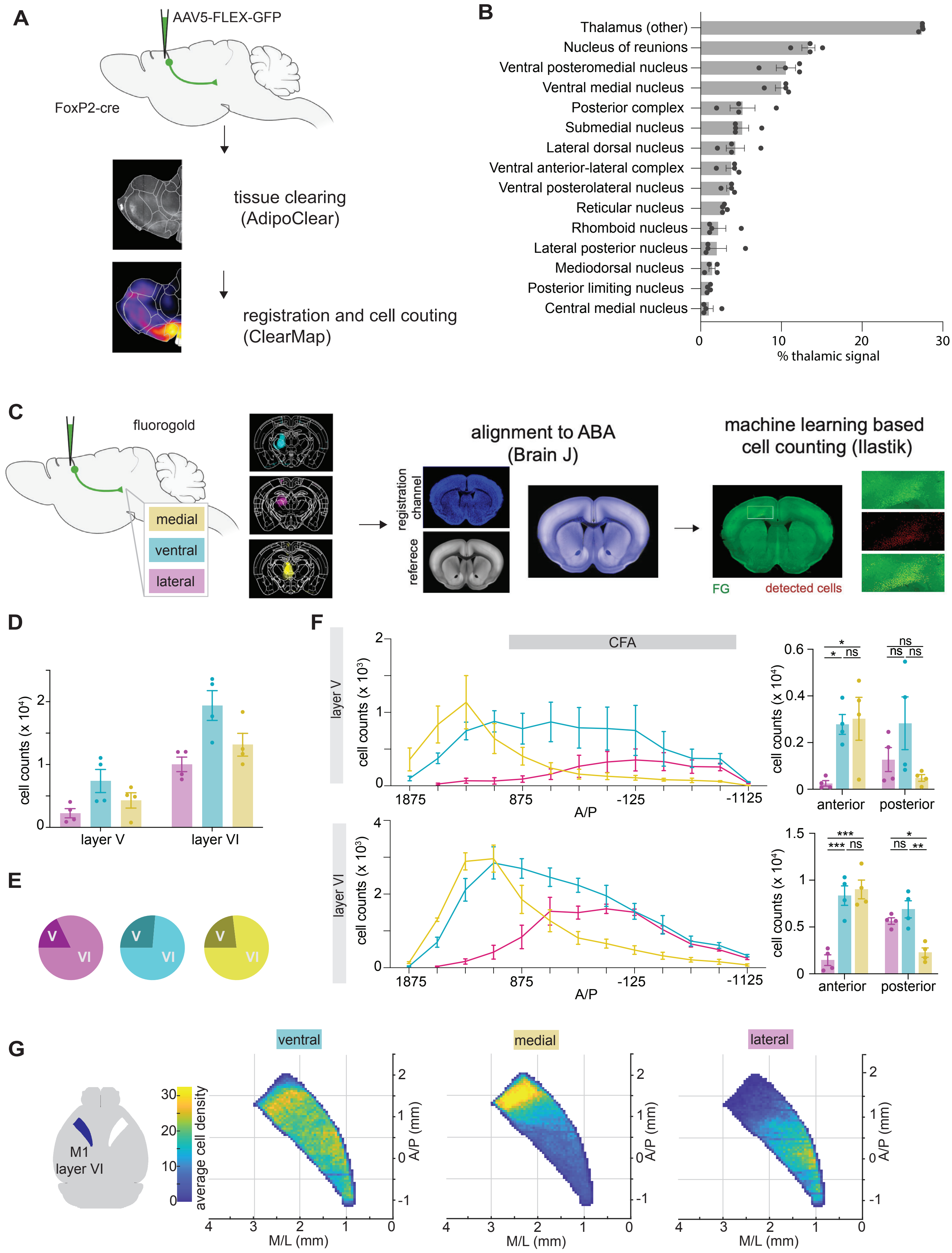
A
AAV5-FLEX-GFP
FoxP2-cre
tissue clearing (AdipoClear)
registration and cell couting (ClearMap)
B
Thalamus (other)
Nucleus of reunions
Ventral posteromedial nucleus
Ventral medial nucleus
Posterior complex
Submedial nucleus
Lateral dorsal nucleus
Ventral anterior-lateral complex
Ventral posterolateral nucleus
Reticular nucleus
Rhomboid nucleus
Lateral posterior nucleus
Mediodorsal nucleus
Posterior limiting nucleus
Central medial nucleus
0 10 20 30
% thalamic signal
C
fluorogold
medial
ventral
lateral
alignment to ABA (Brain J)
registration channel
referece
machine learning based cell counting (Ilastik)
FG
detected cells
D
cell counts (x 10⁴)
layer V
layer VI
E
V
VI
F
CFA
layer V
layer VI
cell counts (x 10³)
1875 875 -125 -1125
A/P
cell counts (x 10⁴)
anterior
posterior
ns
*
ns
***
**
G
ventral
medial
lateral
M1 layer VI
average cell density
M/L (mm)
A/P (mm)

Figure S3. Topographical organization and projection pattern of M1^{CT} neurons

A Schematic of M1^{CT} projection mapping approach in thalamus from M1 in FoxP2-cre mice. Max projection of representative M1^{CT} projections in thalamus; mCherry labeling. Heatmap of thalamic signal; N=4. **B** Thalamic regions with greater than 1% of total thalamic signal; N=4; error bars, SEM. **C** Schematic of retrograde labeling of M1^{CT} neurons from three regions of thalamus: medial (yellow), ventral (cyan), and lateral (magenta). Heatmap of injection site, N=4 animals for all analysis (C-G) and color corresponds to region targeted (C-G). Subsequent workflow consisted of alignment and segmentation using BrainJ and Ilastik. **D** Number of M1^{CT} neurons labeled from each thalamic region in both M1 layer V and layer VI; see Supplementary Table 3 for summary of statistics. **E** Percent of total signal in either cortical layer; **F** Left, Number of M1^{CT} neurons labeled in layer V (top) or layer VI (bottom) from each thalamic region along the anterior/posterior axis; 0=Bregma; error bars, SEM. Right, aggregate cell counts of M1^{CT} neurons labeled from each thalamic target in anterior (1625 to 875 um A/P) or posterior (125 to -375 um A/P) regions of M1; error bars, SEM; for layer VI: two-way ANOVA, p=5.55x10-6 (anterior, medial v. lateral); p=0.0173 (posterior, medial v. lateral); see Supplementary Table 3 for summary of statistics. **G** Distribution of cell density of layer VI M1^{CT} from each thalamic target; left, schematic of M1 layer VI, left hemisphere, blue, mapped. Panels A and C were created in BioRender. Carmona, L. (2025) https://BioRender.com/c14e108.

Figure S4

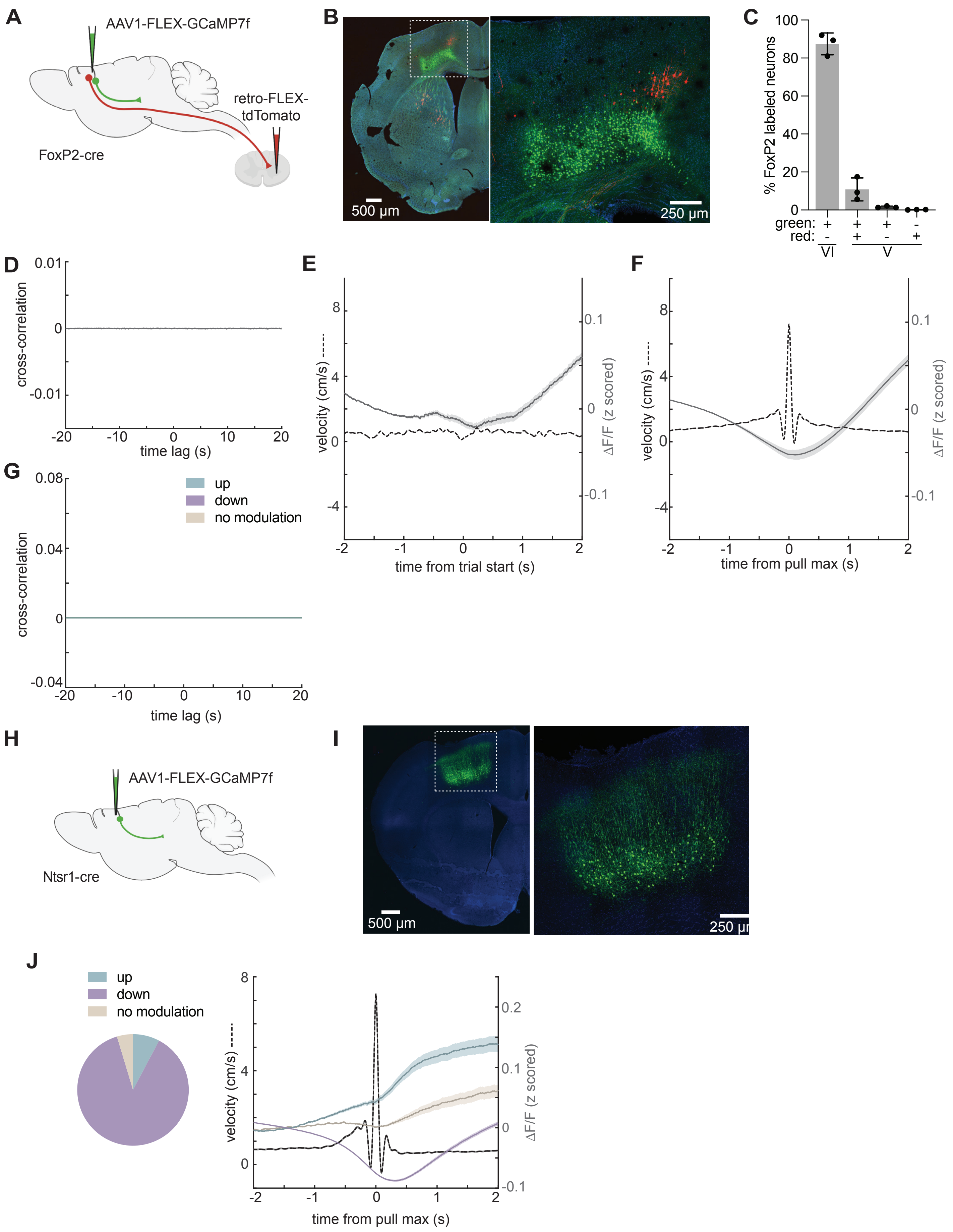

A
AAV1-FLEX-GCaMP7f
retro-FLEX-tdTomato
FoxP2-cre
B
500 µm
250 µm
C
% FoxP2 labeled neurons
100
80
60
40
20
0
green: + + + -
red: - + - +
VI
V
D
cross-correlation
0.01
0
-0.01
-20 -10 0 10 20
time lag (s)
E
velocity (cm/s)
ΔF/F (z scored)
8
4
0
-4
0.1
0
-0.1
-2 -1 0 1 2
time from trial start (s)
F
velocity (cm/s)
ΔF/F (z scored)
time from pull max (s)
G
up
down
no modulation
cross-correlation
0.08
0.04
0
-0.04
time lag (s)
H
AAV1-FLEX-GCaMP7f
Ntsr1-cre
I
500 µm
J
up
down
no modulation
velocity (cm/s)
ΔF/F (z scored)
0.2
0.1
0
-0.1
time from pull max (s)

Figure S4. Calcium imaging approach and M1^{CT} activity

A Schematic of injection strategy for calcium imaging of M1^{CT} neurons in FoxP2-cre mice. An AAV expressing cre-dependent GCaMP7f was injected into the caudal forelimb area of M1. A retrograde AAV expressing cre-dependent tdTomato was also injected into the cervical spinal cord to allow for exclusion of FoxP2 expressing corticospinals from imaging fields. **B** Antibody amplified GCaMP7f signal, green; DAPI, blue; corticospinal neurons, red. **C** Percent of all FoxP2 labeled neurons that are single positive (GCaMP7f, layer VI, 87.5 +/- 5.73), double positive (GCaMP7f and retrograde, cre-dependent TdTomato injected into cervical spinal cord, layer V, 10.8 +/- 6.05); single positive (GCaMP7f, layer V, estimated, 1.56 +/- 0.516); single positive (cre-dependent TdTomato injected into cervical spinal cord, layer V, 0.156 +/- 0.0784); N=3 animals. **D** Average cross-correlation of 100 iterations of shuffled Z-scored calcium ∆F/F for each unit to wheel velocity for whole session; mean, solid gray line; shaded area, SEM. **E,F** Z-scored ∆F/F during unsuccessful trials **(E)** OR wheel pulls **(F)** mean, solid gray line; shaded area, SEM; Wheel velocity (cm/s), dotted line; n=1078 units; N=4 animals for **(D-F)**. **G** Average cross-correlation of 100 iterations of shuffled Z-scored calcium ∆F/F for each unit to wheel velocity for whole session by group; solid lines, mean; shaded areas, SEM; n=740 downmodulated units, n=168 upmodulated units, n=168 non-modulated units; N=4 animals. **H** Schematic of injection strategy for calcium imaging of M1CT neurons in Ntsr1-cre mice. **I** Antibody amplified GCaMP7f signal, green; DAPI, blue; corticospinal neurons, red**.** **J** Left, classification of all units across sessions based on activity during wheel pulls in Ntsr1-cre animals. Right, Z-scored ∆F/F of each group during wheel pulls; solid lines, mean; shaded areas, SEM; wheel velocity during pulls (cm/s), dotted line; 0=pull velocity max; n= 1052 downmodulate units, n=93 upmodulated units, n=56 no modulation units. Panels A and H were created in BioRender. Carmona, L. (2025) https://BioRender.com/c38u586.

Figure S5

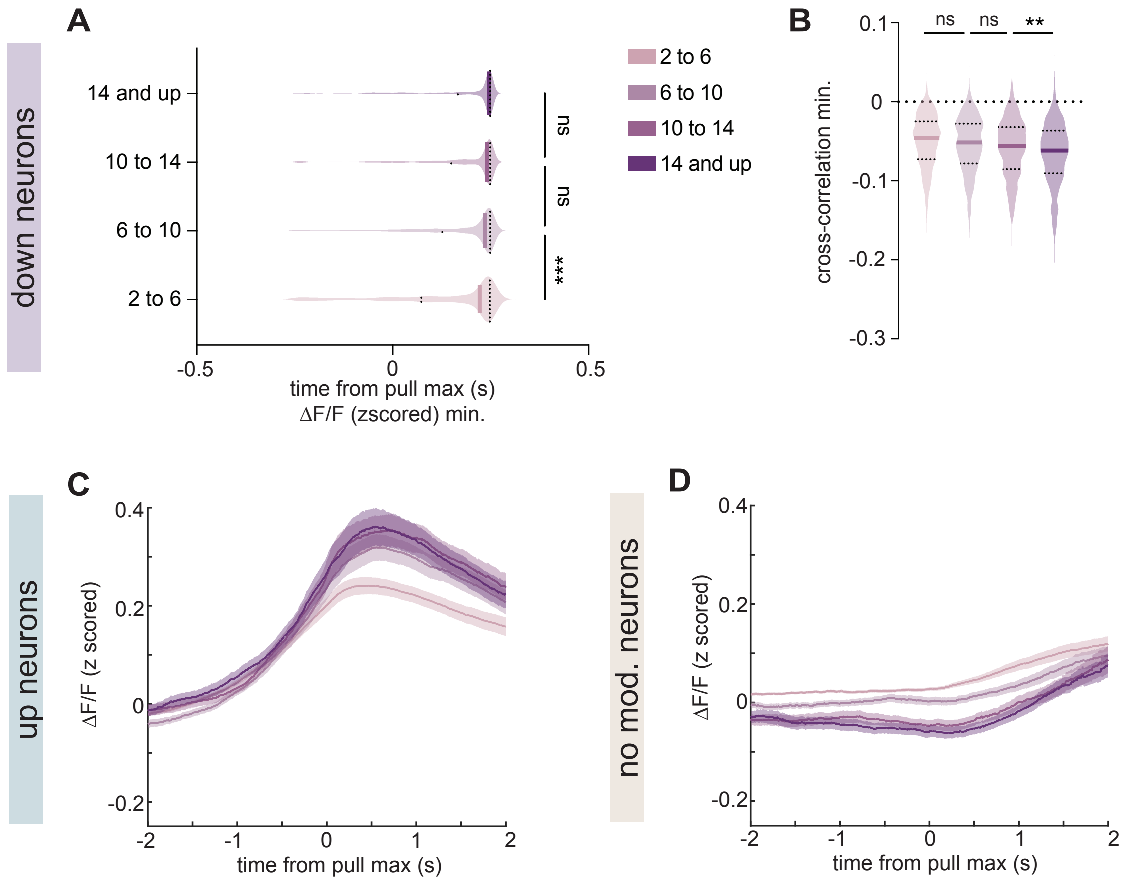

Figure S5. Features of M1[CT] activity during pulls of varying velocity

As defined by classification, units with decreases, n=740 units **(A,B)**, increases, n=168 **(C)**, or no modulation, n=168 **(D)** in activity during wheel pulls; legend for all as in A, wheel velocity (cm/s). All from N=4 animals. **A,B** Distribution of time of ΔF/F minimum **(A)** or minimum of cross-correlation of wheel velocity to z-scored ΔF/F **(B)** during pulls of increasing velocity; thick, line, mean; thin lines, quartiles; One way ANOVA, multiple comparisons, p=3.63x10-4, 2 to 6 v. 6 to 10; p=0.1759, 6-10 v. 10-14; p=0.8196, 10-14 v. 14 and up **(A)**. One way ANOVA, multiple comparisons, p=0.0711, 2-6 v. 6 to 10; p=0.0657, 6-10 v. 10-14; p=0.0.039, 10-14 v. 14 and up **(B)**. See Supplementary Table 3 for p-values for all other comparisons and summary of statistics. **C,D** Z-scored ΔF/F during pulls of increasing velocity; solid line, mean; shaded area, SEM.

Figure S6

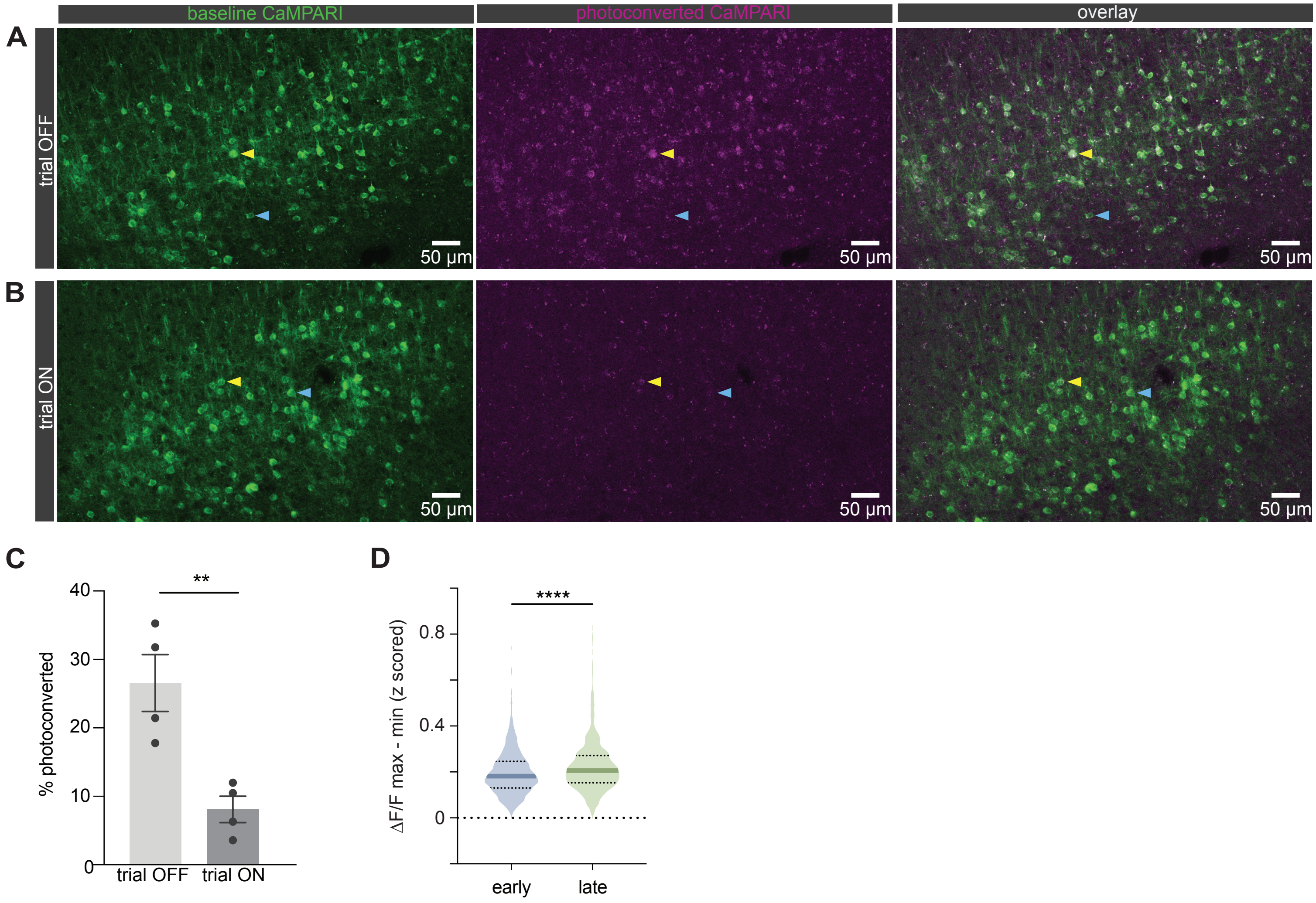

Figure S6. Closed loop labeling of CaMPARI in M1^{CT} neurons

A, B Representative images of CaMPARI labeling in M1^{v} outside of trials (A; trial OFF) or during trials (B, trial ON). Green, baseline, left; red, photoconverted state, middle. Right panels are overlays of both channels. Yellow arrows, cells with photoconverted CaMPARI; Blue arrows, cells with no detectable CaMPARI photocoversion. **C** Percent of CaMPARI labeled cells with photoconversion signal in either trial OFF or trial ON closed-loop labeling; error bars, SEM; two-tailed unpaired t-test, p=0.0068. N=4 animals for each condition. **D** Distribution of the difference in z-scored ΔF/F maximum and minimum before and during wheel pulls in down modulated neurons at early and late sessions; t-test, p=1.75x10-8; early: n=231 units, late: n= 228 units from N=4 mice.

Figure S7

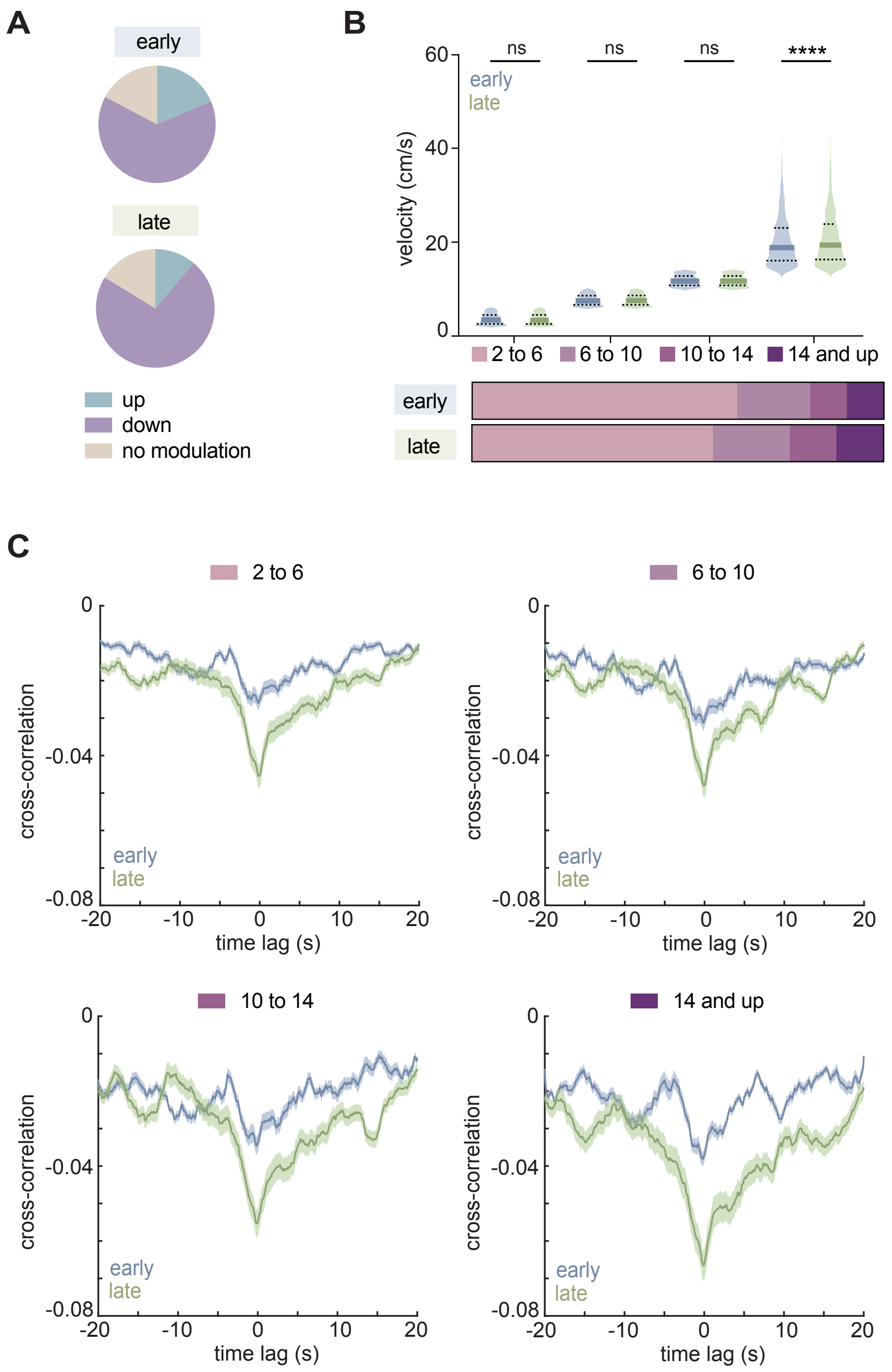
A
early
late
up
down
no modulation
B
ns
ns
ns
****
early
late
velocity (cm/s)
60
40
20
0
2 to 6
6 to 10
10 to 14
14 and up
early
late
C
2 to 6
6 to 10
10 to 14
14 and up
cross-correlation
0
-0.04
-0.08
-20
-10
0
10
20
time lag (s)
early
late

Figure S7. Comparison of M1^{CT} activity during early and late training

A Classification of all units over early and late sessions based on activity during wheel pulls. For early, n=237 down modulated units, n=69 up modulated units, n=64 no modulation units; for late, n=225 down modulated units, n=35 up modulated units, n=50 no modulation units. All from N=4 animals. **B** Top, distribution of peak velocities (cm/s) in pulls of each velocity category in early (blue) or late (green) sessions; thick line, mean; thin lines, quartiles. One-way ANOVA, multiple comparisons, for early v. late, p=989, 2-6; p>0.999, 6-10 and 10-14; p<1x10-15, 14 and up; see Supplementary Table 3 for p-values for all other comparisons and summary of statistics; 2 to 6 early, n=8910 trials; 2 to 6 late, n=11150 trials; 6 to 10 early, n=2451 trials; 6 to 10 late, n=3555 trials; 10 to 12 early, n=1235 trials; 10 to 14 late, n=2161 trials; 14 and up early, n=1240 trials; 14 and up late, n=2168 trials. All from N=4 mice. Bottom, proportion of pulls in early or late sessions in each velocity category. **C** Cross-correlation of wheel pulls for early (blue) or late (green) training sessions of down modulated neurons to encoder velocity, grouped by increasing pull velocity (cm/s); solid line, mean; shaded area, SEM; early: n=231 units, late: n=228 units from N=4 mice.

Figure S8

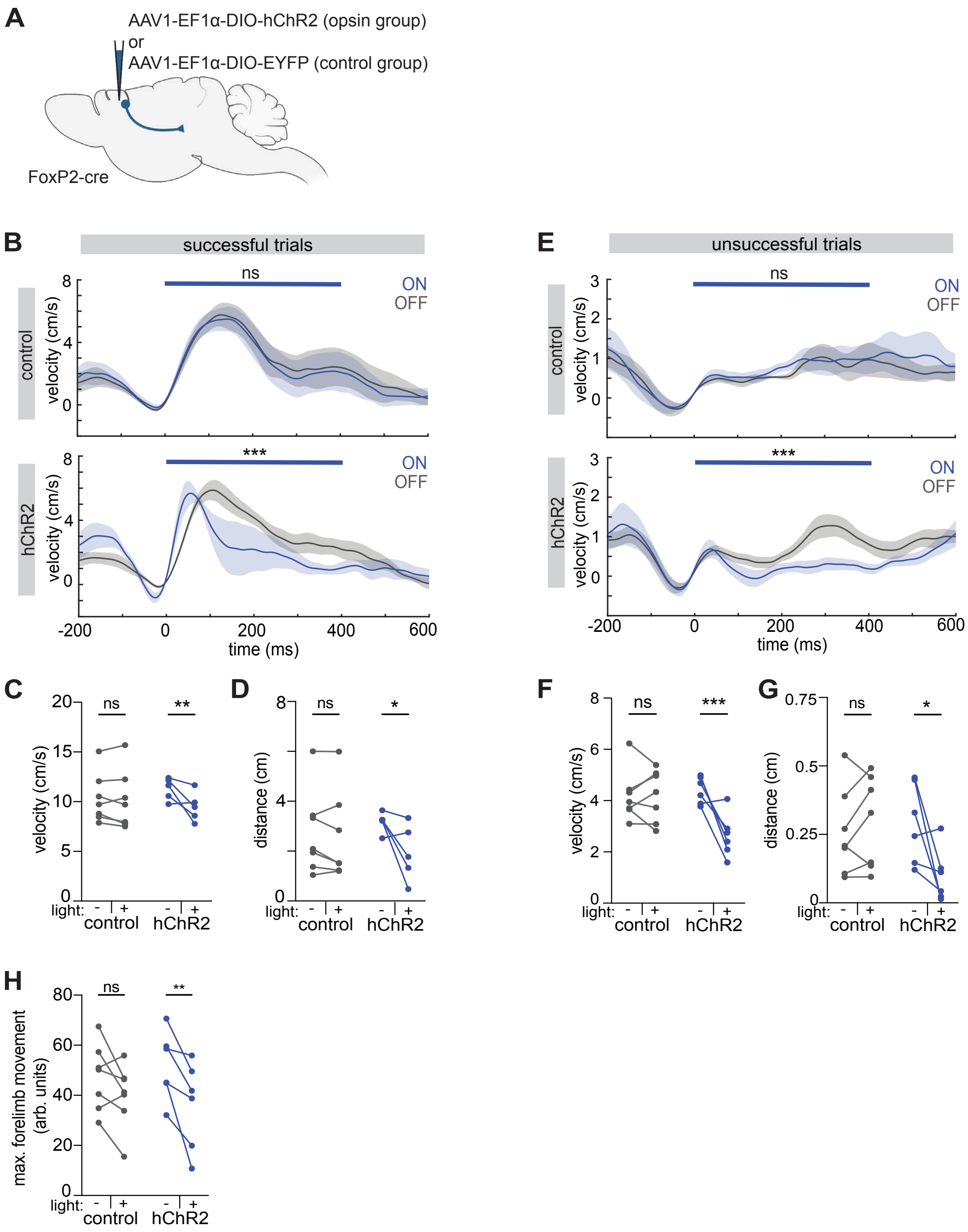

A
AAV1-EF1α-DIO-hChR2 (opsin group)
or
AAV1-EF1α-DIO-EYFP (control group)
FoxP2-cre
B
successful trials
control
hChR2
velocity (cm/s)
ns
***
ON
OFF
time (ms)
E
unsuccessful trials
C
D
F
G
H
velocity (cm/s)
distance (cm)
max. forelimb movement (arb. units)
light:
control
hChR2
ns
**
*

Figure S8. Closed-loop optogenetic perturbation at trial initiation

A Schematic of injection strategy for optogenetic experiments targeting $M1^{CT}$ neurons; created in BioRender. Carmona, L. (2025) https://BioRender.com/b23i558. **B,E** Velocity traces (cm/s) of successful **(B)** and unsuccessful **(E)** trials from control, top, and hChR2, bottom, animals during light, blue, and no light trials, gray, of closed-loop with light delivery at trial start; 0=trial start; blue bar denotes time of light; p>0.999 (control), p<1x10-15 (hChR2) **(B)** and **(E)**, time x condition, two-way ANOVA; solid line, mean; shaded area, SEM. **C,F** Max velocity (cm/s) of successful trials **(C)** and unsuccessful trials **(F)** for each animal with light on or off in each group; two way ANOVA, multiple comparisons. C, p=0.905 (control), p=0.0098 (hChR2), p=0.0327 for group x condition; F, p>0.999 (control), p=0.0010 (hChR2), p=0.0044 for group x condition; **D,G** Wheel distance (cm) traveled during successful **(D)** and unsuccessful **(G)** trials for each animal with light on or off for each group, D, two way ANOVA, multiple comparisons, p=0.865 (control), p=0.0132 (hChR2), p=0.0451 for group x condition; G, p=0.751 (control), p=0.0145 (hChR2), p=0.0149 for group x condition. **H** Max distance traveled by right forelimb during trials; two-way ANOVA, multiple comparisons; p=0.195, control; p=0.0088, hChR2; p=0.186 for group x condition; N=7 animals, control group; N=6 animals, hChR2 group for B-H. Note that an animal with no successful trials with light on has been removed from B-D. See Supplementary Table 3 for summary of all statistics.

Figure S9

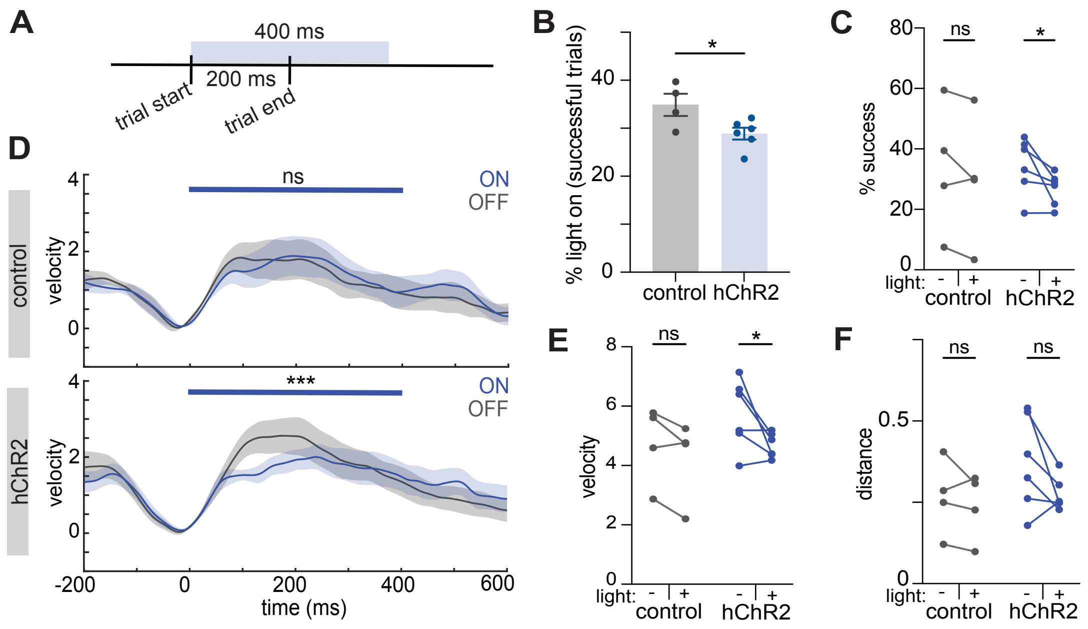

Figure S9. Optogenetic manipulations in Ntsr1-cre mice

A Schematic of optogenetic light delivery for closed loop at trial start at late training for wheel turning task in Ntsr1-cre animals. Light was pulsed at 20 hz; 10 ms pulse width. **B** Percent of successful trials with light on (# successful trials during light on / # of all successful trials); control, gray; hChR2, blue; error bars, SEM; two-tailed unpaired t-test, p=0.0357. For B-F: N=4 animals, control group; N=6 animals, hChR2 group. Each point is average of all light delivery sessions. **C** Percentage of trials that are successful for each animal with light on or off in each group (# successful trials during light on(off) / # of all trials during light on(off)); error bars, SEM; two-way ANOVA, multiple comparisons; p=0.530, control; p=0.049, hChR2, p=0.389 for group x condition. **D** Velocity traces of all trials from control, top, and hChR2, bottom, animals during light, blue, and no light trials, gray; 0=trial start; blue bar denotes time of light, p>0.999 (control), p=8.60x10-7 (hChR2), time x condition, two way ANOVA; solid line, mean; shaded area, SEM. **E** Maximum velocity of all trials for each animal with light on or off in each group; two-way ANOVA, multiple comparisons, p=0.549 (control), p=0.0452 (hChR2), p=0.360 for group x condition; **F** Wheel distance traveled during all trials for each animal with light on or off for each group; two way ANOVA, multiple comparisons, p=0.862, control, p=0.0997, hChR2, p=0.320 for group x condition. See Supplementary Table 3 for summary of all statistics.

Figure S10

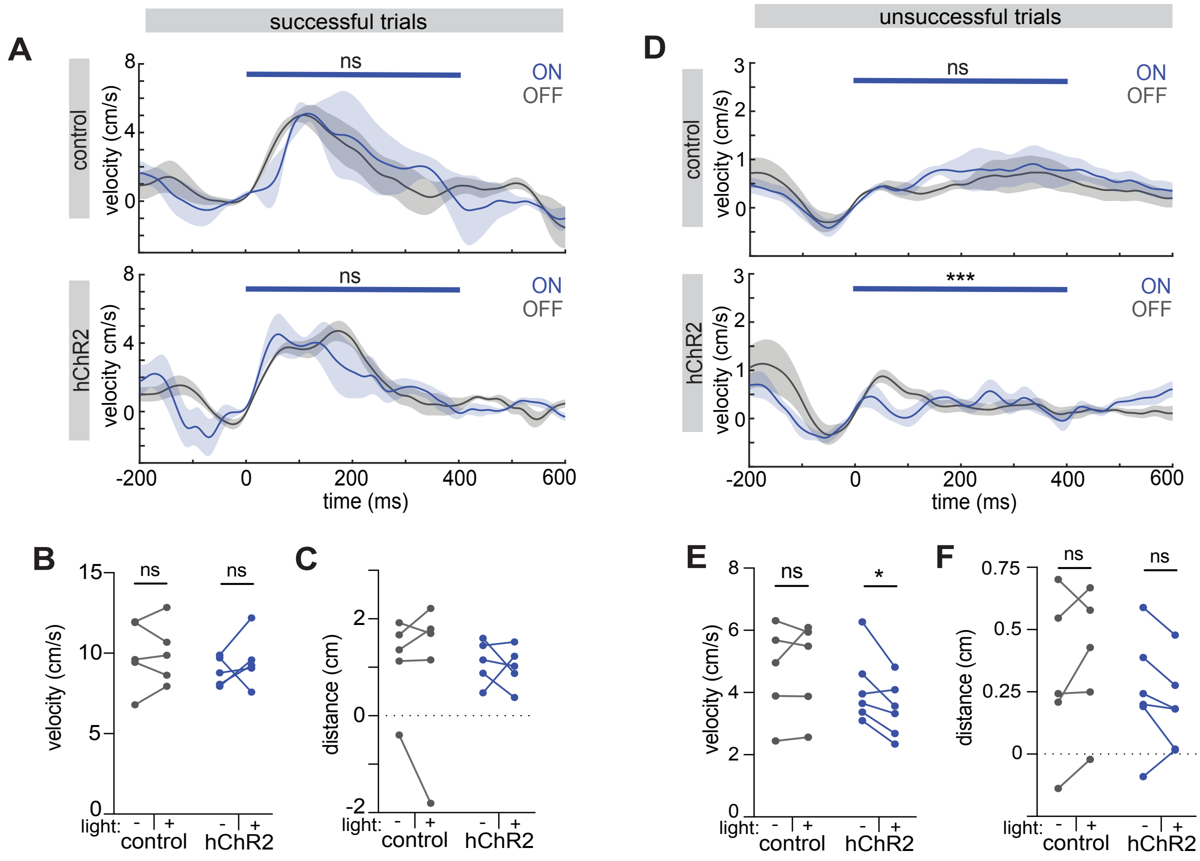

Figure S10. Optogenetic manipulations during early training

A,D Velocity traces (cm/s) of successful **(A)** and unsuccessful **(D)** trials from control, top, and hChR2, bottom, animals during light, blue, and no light trials, gray, of closed-loop with light delivery at trial start; 0=trial start; blue bar denotes time of light; p>0.999 (control), p>0.999 (hChR2) (A), p>0.999 (control), p<1x10-15 (hChR2) D, time x condition, two-way ANOVA; solid line, mean; shaded area, SEM. **B,E** Max velocity (cm/s) of successful trials **(B)** and unsuccessful trials **(E)** for each animal with light on or off in each group; two way ANOVA, multiple comparisons, B, p=0.996 (control), p=0.552 (hChR2), p=0.520 for group x condition; E, p=0.844, (control), p=0.0316 (hChR2), p=0.0405 for group x condition; **C,F** Wheel distance (cm) traveled during successful **(C)** and unsuccessful **(F)** trials for each animal with light on or off for each group; two way ANOVA, multiple comparisons, C, p=0.903, (control), p=0.933, hChR2, p=0.960 for group x condition; F, p=0.376 (control), p=0.393 (hChR2), p=0.0922 for group x condition; N=5 animals, control group; N=5 animals, hChR2 group for A-C. N=5 animals, control group; N=6 animals, hChR2 group for D-F. See Supplementary Table 3 for summary of all statistics.

Figure S11

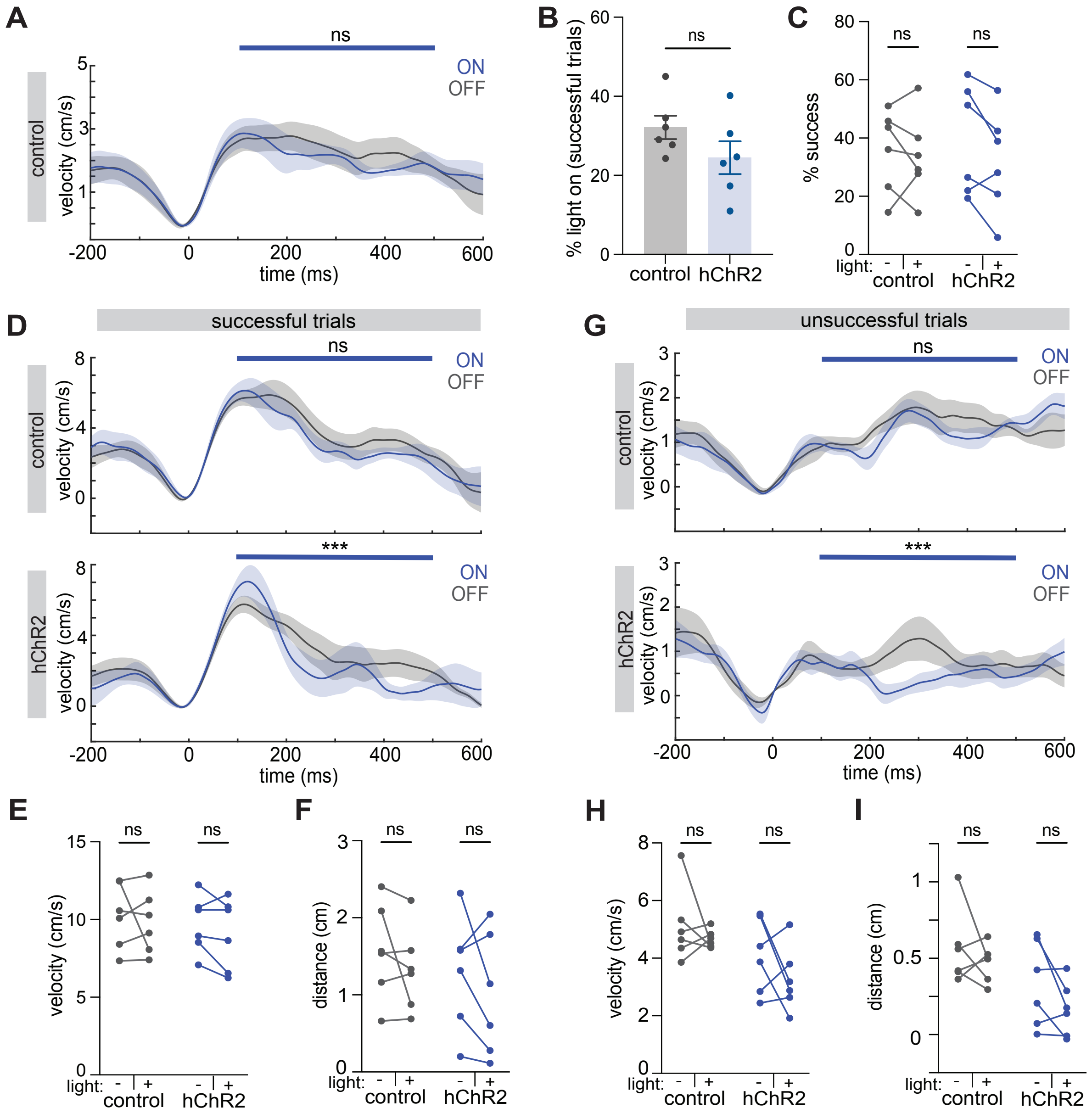

A
ns
ON
OFF
control
velocity (cm/s)
time (ms)
B
% light on (successful trials)
ns
control
hChR2
C
% success
ns
ns
light:
control
hChR2
D
successful trials
ns
***
ON
OFF
control
hChR2
velocity (cm/s)
time (ms)
G
unsuccessful trials
ns
***
ON
OFF
control
hChR2
velocity (cm/s)
time (ms)
E
velocity (cm/s)
F
distance (cm)
H
velocity (cm/s)
I
distance (cm)
ns
light:
control
hChR2

Figure S11. Optogenetic manipulations of ongoing trials

A Velocity traces (cm/s) of control animals during light, blue, and no light trials, gray, of closed-loop light delivery at trial maximum; 0=trial start; blue bar denotes time of light; p=0.862, time x condition, two way ANOVA solid line, mean; shaded area, SEM. See Supplementary Table 3 for summary of statistics. **B** Percent of successful trials with light on, (# successful trials during light on / # of all successful trials); control, gray; hChR2, blue; error bars, SEM; two-tailed unpaired t-test, p=0.164. **C** Percentage of trials that are successful for each animal with light on or off in each group, (# successful trials during light on (OR off) / # of all trials with light on (OR off)); two-way ANOVA, multiple comparisons; p=0.842, control; p=0.147, hChR2. For B-I:N=6 animals, both groups. **D,G** Velocity traces (cm/s) of successful **(D)** and unsuccessful **(G)** trials from control, top, and hChR2, bottom, animals during light, blue, and no light trials, gray, of closed-loop light delivery to max of trial; 0=trial start; blue bar denotes time of light; p=0.433 (control), p<1x10-15 (hChR2) (D), p>0.9999 (control), p<1x10-15 (hChR2) (G), time x condition, two-way ANOVA solid line, mean; shaded area, SEM. **E,H** Max velocity (cm/s) of successful trials **(E)** and unsuccessful trials **(H)** for each animal with light on or off in each group; two way ANOVA, multiple comparisons. E, p=0.808 (control), p=0.602 (hChR2), p=0.815 for group x condition; H, p=0.7456 (control), p=0.379 (hChR2), p=0.667 for group x condition; **F,I** Wheel distance (cm) traveled during successful **(F)** and unsuccessful **(I)** trials for each animal with light on or off for each group; error bars, SEM; two way ANOVA, multiple comparisons, F, p=0.523, (control), p=0.394 (hChR2), p=0.872 for group x condition; I, p=0.597 (control), p=0.231 (hChR2), p=0.614 for group x condition; See Supplementary Table 3 for summary of all other statistics.

Figure S12

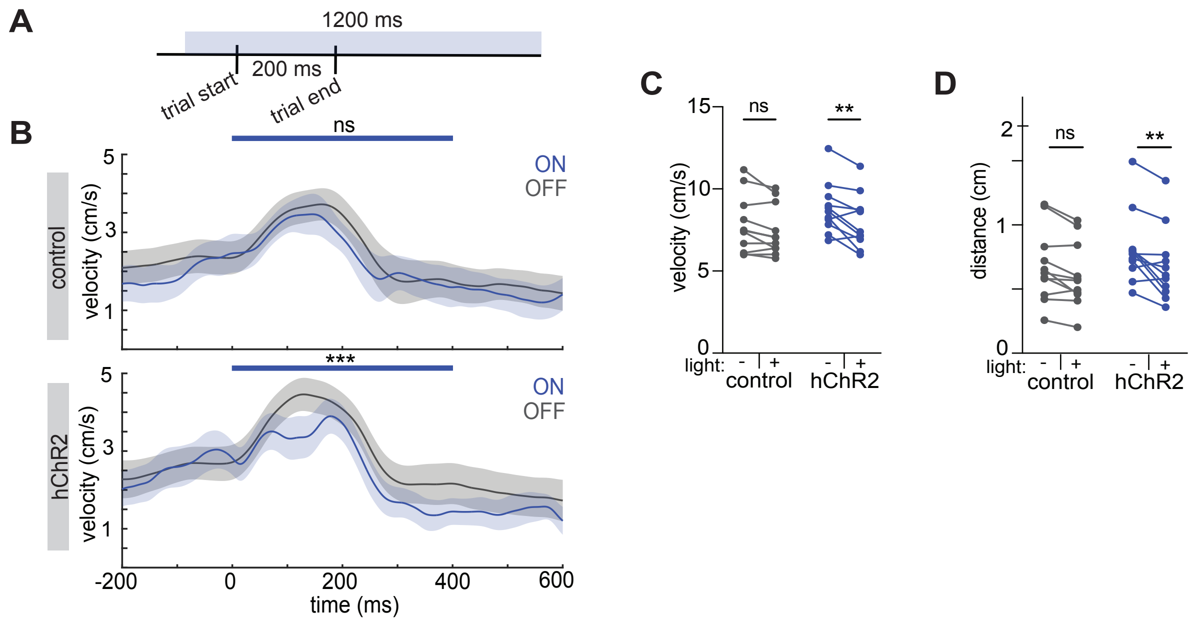

Figure S12. Optogenetic manipulations prior to trial start

A Schematic of optogenetic light delivery for open loop trials with light preceding pull start. **B** Velocity traces (cm/s) of open loop trials with light preceding pull from control, top, and hChR2, bottom, animals during light, blue, and no light trials, gray; 0=trial start; blue bar denotes time of pull equivalent to closed loop trials, $p>0.999$ (control), $p=5.29 \times 10^{-10}$ (hChR2), time x condition, two way ANOVA; solid line, mean; shaded area, SEM. Note that light is on for full duration of trace in light on trials as depicted in A. for B-D: N=10 animals for control group and N=11 animals for hChR2 group. **C** Max velocity (cm/s) of open loop trials with light preceding pull for each animal with light on or off in each group; two way ANOVA, multiple comparisons, $p=0.156$ (control), $p=0.0016$ (hChR2), $p=0.151$ for group x condition. **D** Wheel distance (cm) traveled during open loop trials with light preceding pull for each animal with light on or off for each group; error bars, SEM; two way ANOVA, multiple comparisons, $p=0.0708$ (control), $p=0.0011$ (hChR2), $p=0.202$ for group x condition. See Supplementary Table 3 for summary of all other statistics.

Figure S13

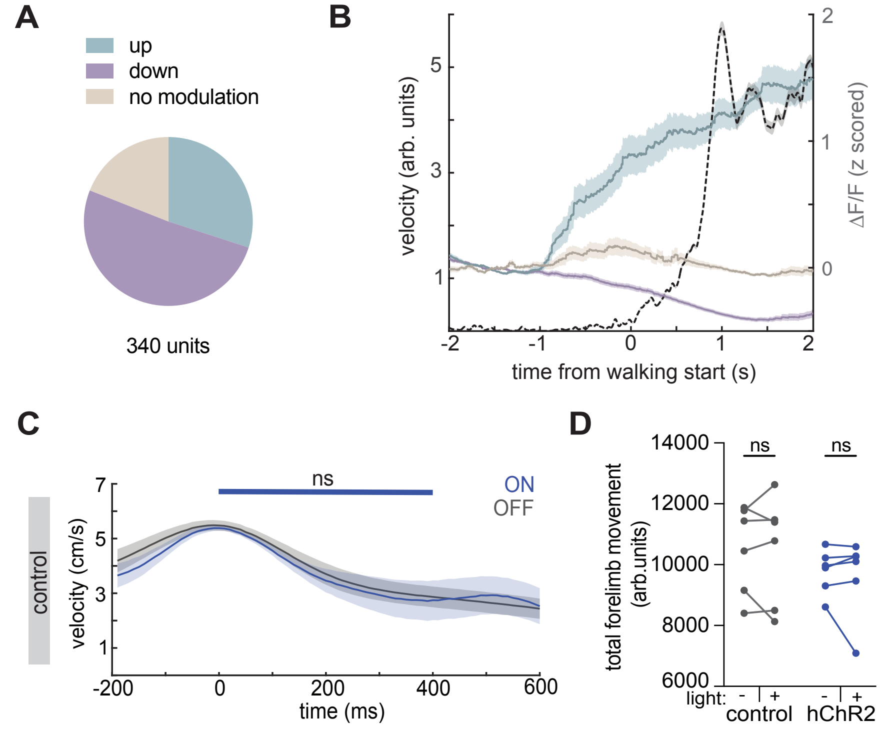

Figure S13. Optogenetic manipulations during walking

A Classification of all units across sessions based on activity during walking bouts. **B** Z-scored ΔF/F of each group during walking bouts; solid lines, mean; shaded areas, SEM; wheel velocity (arbitrary units), dotted line; 0=start of walking bout. For A,B, n=173 downmodulated units, n=102 upmodulated units, n=65 no modulation units; N=3 animals. **C** Velocity traces (cm/s) of walking trials from control animals (see Figure 4F for hChR2 animals) during light, blue, and no light trials, gray; 0=trial start; blue bar denotes time of light, $p>0.999$ (control), $p=0.996$ (hChR2), time x condition, two way ANOVA, See Supplementary Table 3 for summary of statistics; solid line, mean; shaded area, SEM. **D** Total distance traveled by right forelimb during walking trials, two-way ANOVA, multiple comparisons; $p=0.992$ and $p=0.848$ for control and hChR2, respectively, $p=0.776$ for group x condition; See Supplementary Table 3 for summary of statistics. N=6 animals both groups.

Figure S14

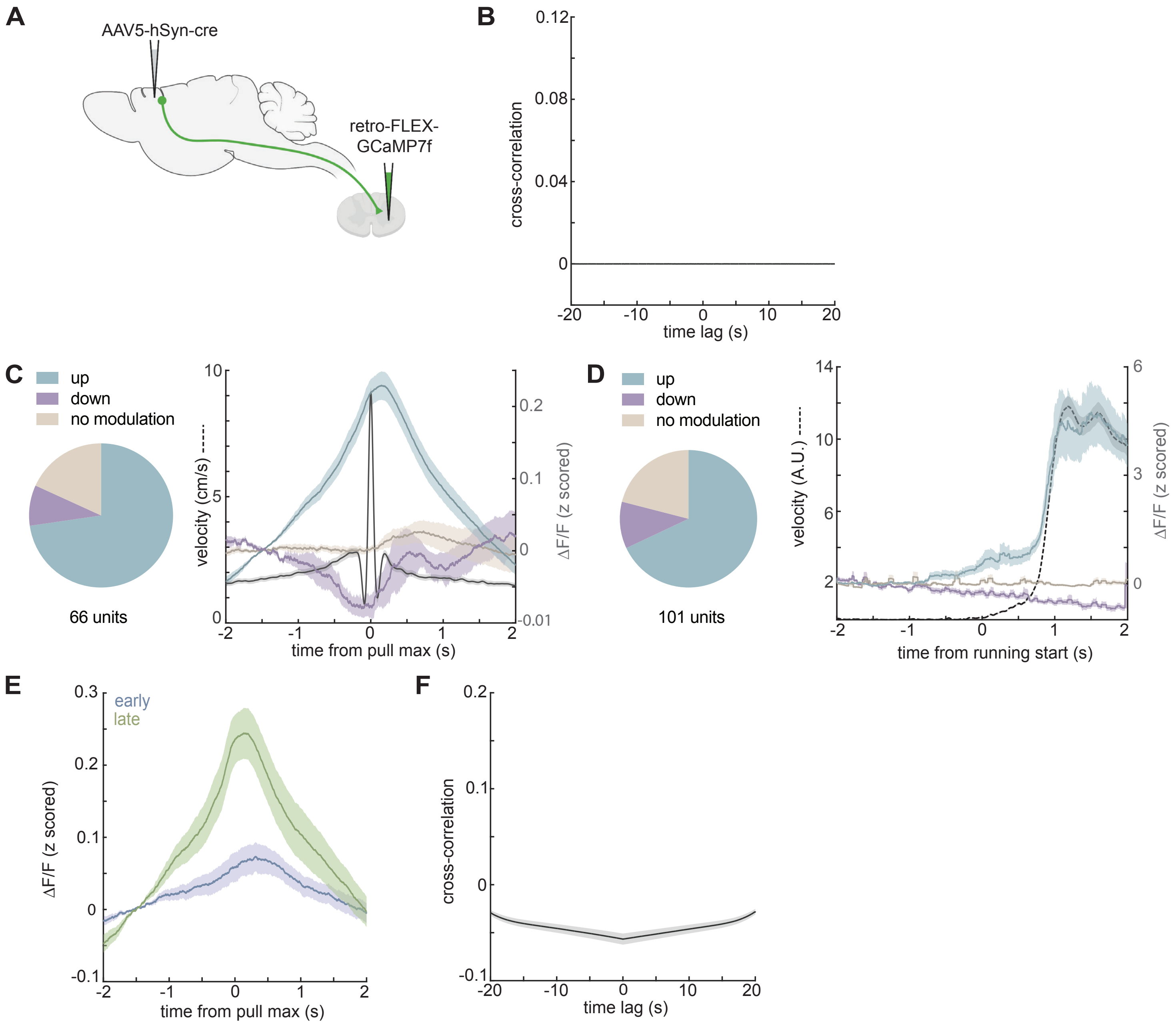

Figure S14. Calcium imaging of M1 corticospinal activity

A Schematic of injection strategy for calcium imaging of M1 corticospinal neurons; created in BioRender. Carmona, L. (2025) https://BioRender.com/z58q140. **B** Average cross-correlation of 100 iterations of shuffled Z-scored calcium ΔF/F for each unit over the whole session to wheel velocity for whole session; mean, solid gray line; shaded area, SEM; n=66 units from N=3 mice. **C,D.** Classification of all units across sessions based on activity during wheel pulls, n=48 upmodulated units, n=6 downmodulated units, n=12 no modulation units. **(C)** or during locomotion, n=74 upmodulated units, n=12 downmodulated units, n=23 no modulation units **(D)**. All from N=3 mice. Right, Z-scored ΔF/F of each group; solid lines, mean; shaded areas, SEM; wheel velocity during pulls (cm/s) or during walking (arbitrary units) dotted line; 0=pull velocity max. **E** Z-scored ΔF/F during wheel pulls at early, blue, or late, green, training, n=20 units, early; n=22 units, late; N=3 animals. **F** Average cross-correlation of 100 iterations of shuffled Z-scored calcium ΔF/F for each unit during wheel pulls to wheel velocity during wheel pulls; solid line, mean; shaded areas, SEM; n=66 units from N=3 mice.

Figure S15

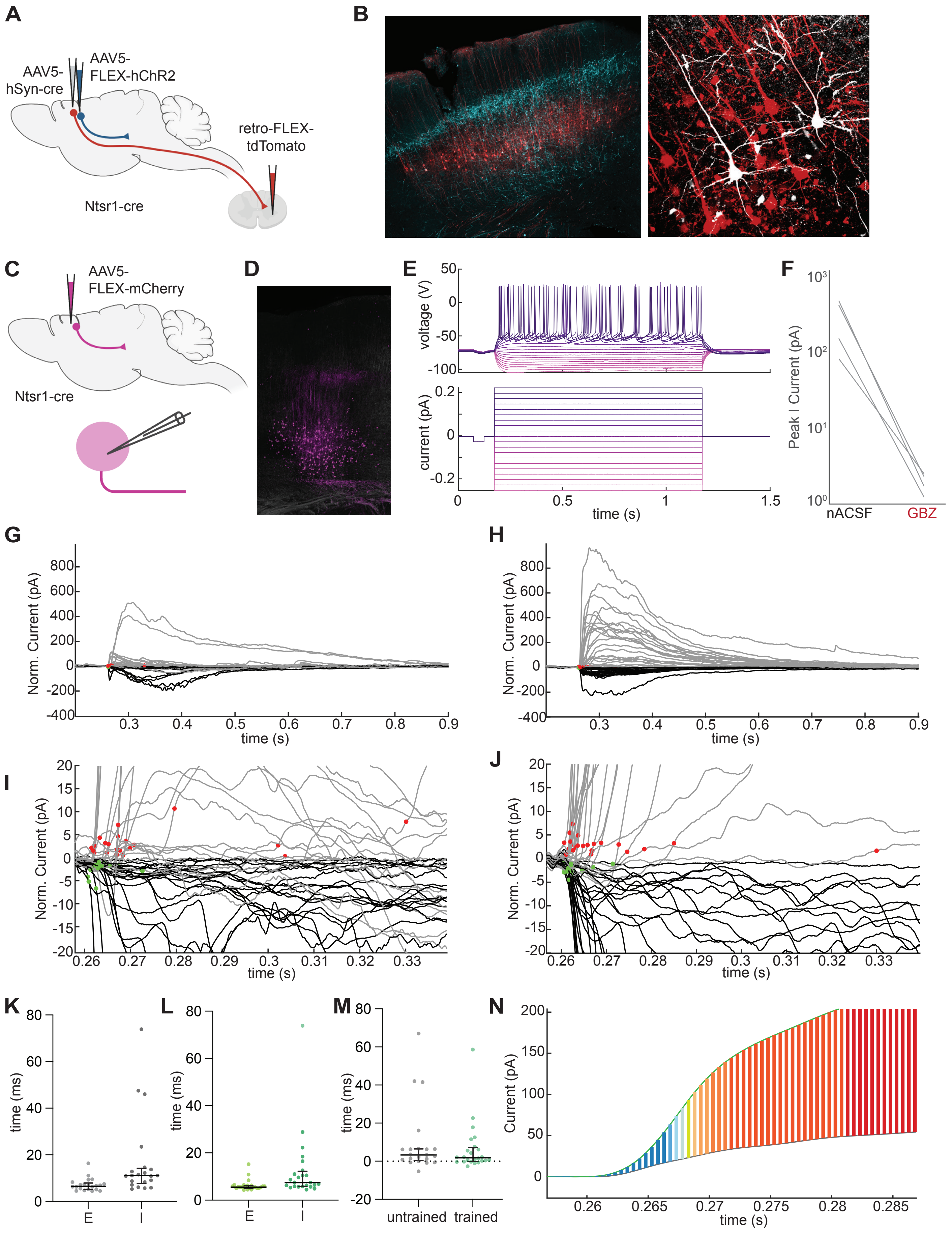

A
AAV5-
hSyn-cre
AAV5-
FLEX-hChR2
retro-FLEX-
tdTomato
Ntsr1-cre
B
C
AAV5-
FLEX-mCherry
Ntsr1-cre
D
E
voltage (V)
current (pA)
time (s)
F
Peak I Current (pA)
nACSF
GBZ
G
H
Norm. Current (pA)
time (s)
I
J
K
L
M
time (ms)
E
I
untrained
trained
N
Current (pA)
time (s)

Figure S15. Slice electrophysiology of M1 corticospinal neurons during M1^{CT} photostimulation

A Schematic of injection strategy for labeling M1 corticospinal neurons and expressing channelrhodopsin (hChR2) in M1^{CT} neurons in Ntsr1-cre mice. **B** Expression of hChR2 (cyan) in M1^{CT} neurons and tdTomato in corticospinal neurons (red), top; Neurobiotin filled corticospinals post recording (white). **C** Peak inhibitory current with control treatment (nACSF) or GABAzine (GBZ); p=0.0284, paired t-test; N=4 units. **D** Schematic of injection strategy for labeling M1^{CT} neurons in Ntsr1-cre mice, top, for recordings of baseline firing properties, bottom. **E** Expression of mCherry in M1^{CT} neurons. **F** Firing of exemplar M1^{CT} neuron, top, at varying currents, bottom. **G-J** Responses of corticospinal neurons after stimulation of corticothalamic neurons; smoothed 2ms sliding window. Excitatory, black; inhibitory, gray. **I, J** zoom in of **G, H,** respectively; dots indicate time when response is at least 3 SD above the baseline period; green excitatory; red, inhibitory. **K,L** Quantification of latency of excitatory and inhibitory responses post stimulus. **K,** untrained; **L,** trained; horizontal line, median; vertical line, IQR. **M** Difference in inhibitory and excitatory latencies (IPSC - EPSC per cell) in untrained, gray, or trained, green, animals; horizontal line, median; vertical line, IQR. **N** Comparison of inhibitory responses in neurons from untrained, lower gray line, and trained, upper green line, binned over 0.5 ms. Each bar denotes p-value of each set of values at that timepoint. Light green bar, first significant timepoint; For G-N, n=22 units, N=2 mice, untrained and n=24 units, N=2 mice, trained. Panels A and C were created in BioRender. Carmona, L. (2025) https://BioRender.com/m45s893.

Supplementary Table 1

summary metrics	
median UMI count	7.97E+03
median gene count	3503
total cell count	16490
# runs	
control	4
early training	4
late training	5

	control	early	late
median UMI count	8557	6198	10390
median gene count	3694	2911	4035
total cell count	5369	5493	2923

Supplementary Table 1. Summary of sc-RNAseq experiments

Summary metrics, number of runs, and metrics by condition for single-cell RNA sequencing runs of CaMPARI labeled neurons in M1.

Supplementary Table 2

Figure 1G												
	ANCOM-BC						MASC					
	pvalue			logfc			pvalue			oddsratio		
	early_vs_control	late_vs_control	late_vs_early	early_vs_control	late_vs_control	late_vs_early	early_vs_control	late_vs_control	late_vs_early	early_vs_control	late_vs_control	late_vs_early
L2/3IT Cux2+_Rorb-	0.314	0.688	0.254	-0.270	-0.087	-0.318	0.416	0.326	0.800	0.833	0.781	0.937
L5/6IT Fezf2+_Ccdc80+	0.385	0.000	0.573	-0.126	0.444	-0.188	0.741	0.010	0.010	0.950	1.417	1.520
L5/6IT Rorb+_Adam33+_Hspb3+	0.900	0.597	0.006	0.027	-0.163	0.070	0.584	0.390	0.147	1.182	0.739	0.636
L5/6IT Rorb+_Atp5o+	0.145	0.009	0.706	1.266	1.439	-0.691	0.117	0.038	0.847	6.135	4.984	0.828
L5/6NP Fezf2+ _Tshz2+	0.426	0.105	0.101	0.245	0.218	-0.327	0.233	0.349	0.327	1.561	1.177	0.706
L5ET Fezf2+_Bcl6+	0.383	0.007	0.706	0.135	0.557	-0.528	0.210	0.118	0.601	1.270	1.421	1.127
L5IT Rorb+_ Adam33+_Trpm4+	0.191	0.680	0.601	-0.403	-0.091	-0.078	0.349	0.412	0.808	0.727	0.826	1.093
L5IT Rorb+_ Atp6v0c+	0.028	0.493	0.490	-0.487	-0.174	-0.189	0.061	0.328	0.755	0.631	0.699	1.116
L6CT Fezf2+_Foxp2+	0.157	0.000	0.216	0.444	1.269	0.324	0.150	0.006	0.056	1.595	3.235	1.978
Ndnf_Chst7	0.006	0.413	0.004	-0.785	0.097	0.800	0.006	0.796	0.005	0.436	0.945	2.169
Ndnf_Zfp804b	0.218	0.002	0.144	0.478	1.071	0.511	0.132	0.037	0.439	2.928	4.544	1.581
Pvalb	0.838	0.501	0.573	-0.048	0.197	0.163	0.647	0.922	0.589	0.743	1.065	1.423
Sst_Gabrd	3.92E-06	3.55E-09	0.766	0.629	0.760	0.050	0.104	0.068	0.899	3.462	3.711	1.072
Sst_Htr1a	0.374	0.640	0.672	-0.462	-0.136	0.244	0.648	0.812	0.944	0.682	0.902	1.071
Sst_Myh8	0.213	0.565	0.355	0.455	0.231	-0.306	0.450	0.983	0.193	1.824	0.972	0.392
Vip_Pkib	0.386	0.331	0.888	0.405	0.414	-0.074	0.098	0.363	0.685	2.139	1.638	0.779
Vip_Sema5b	0.837	0.176	0.228	-0.043	-0.175	-0.214	0.602	0.016	0.127	0.861	0.552	0.627
Vip_Sncg	0.153	0.021	0.490	0.485	0.703	0.136	0.088	0.074	0.864	4.065	3.711	0.913
Vip_Syt9	0.694	6.20E-06	0.011	-0.091	-0.682	-0.673	0.572	0.004	0.022	0.739	0.096	0.129

Supplementary Table 2. Enrichment analysis statistics

Statistics for enrichment analysis conducted with both ANCOM-BC and MASC all cell types as reported in Figure 1F-H.

Supplementary Table 3

Figure	sample size	summary statistics	statistical test	values
1B	N=6		one way repeated measures ANOVA (time)	P=0.0104; SS=7664; DF=17; MS=450.8; F(3.623, 18.11)=4.69
	N=6 (day 4); N=6 (day 12)	mean (day 4)=20.88; mean (day 12)=41.22; mean diff.=-20.45; SE of diff=7.039	one way ANOVA; planned comparison between day 4 and day 12; Sidak's multiple comparisons	adjusted P value=0.0336; t=2.904; DF=5
1C	N=6		one way repeated measures ANOVA (time)	P=0.0182; SS=66.82; DF=17; MS=3.93; F(3.422, 17.11)=4.2
	N=6 (day 4); N=6 (day 12)	mean (day 4)=4.069; mean (day 12)=5.806; mean diff.=--1.737; SE of diff=0.747	one way ANOVA; planned comparison between day 4 and day 12; Sidak's multiple comparisons	adjusted P value=0.0569; t=2.465; DF=5
1G	see Supplementary Table 2 and Methods for description of test and values			
1H	see Supplementary Table 2 and Methods for description of test and values			
2I	N=4; n=740	2 to 6: mean=-0.120, SD=0.082; 6 to 10: mean=-0.165, SD=0.086; 10 to 14: mean=-0.193, SD=0.104; 14 and up: mean=-0.207, SD=0.124	ordinary one way ANOVA; Tukey's multiple comparison test for all comparisons	P<0.001; SS=3.286; DF=3; MS=1.095; F(3, 2956)=108.4; adjusted P values for multiple comparisons: 2 to 6 vs. 6 to 10: P=<0.0001; 2 to 6 vs. 10 to 14: P=<0.0001; 2 to 6 vs. 14 and up: P=<0.0001; 6 to 10 vs. 10 to 14: P=<0.0001; 6 to 10 vs. 14 and up: P=<0.0001; 10 to 14 vs. 14 and up: P=0.0553
2J	N=4; n=740	2 to 6: mean=0.063, SD=0.037; 6 to 10: mean=0.091, SD=0.053; 10 to 14: mean=0.099, SD=0.062; 14 and up: mean=0.099, SD=0.061	ordinary one way ANOVA; Tukey's multiple comparison test for all comparisons	P<0.0001; SS=0.663; DF=3; MS=0.221; F(3, 2956)=76.49; adjusted P values for multiple comparisons: 2 to 6 vs. 6 to 10: P=<0.0001; 2 to 6 vs. 10 to 14: P=<0.0001; 2 to 6 vs. 14 and up: P=<0.0001; 6 to 10 vs. 10 to 14: P=<0.023; 6 to 10 vs. 14 and up: P=<0.0482; 10 to 14 vs. 14 and up: P=0.994
2M	N=4; n=231 (early), n=228 (late)	2 to 6 early: mean=-0.055, SD=0.041; 2 to 6 late: mean=-0.096, SD=0.058; 6 to 10 early: mean=-0.047, SD=0.104; 6 to 10 late: mean=-0.136, SD=0.079; 10 to 14 early: mean=-0.139, SD=0.053; 10 to 14 late: mean=-0.168, SD=0.098; 14 and up early: mean=-0.161, SD=0.069; 14 and up late: mean=-0.2, SD=0.104	ordinary one way ANOVA; Tukey's multiple comparison test for all comparisons	P<0.0001; SS=3.568; DF=7; MS=0.51; F(7,1828)=98.17; adjusted P values for multiple comparisons: P=<0.0001 for all comparisons except the following; 2 to 6 early vs. 6 to 10 early: P<0.999; 6 to 10 late vs. 10 to 14 early: P=0.999; 6 to 10 late vs 14 and up early: P=0.005; 10 to 14 early vs. 10 to 14 late: P=0.0005; 10 to 14 early vs. 14 and up early: P=0.0318; 10 to 14 late vs. 14 and up early, P=0.9582
3B	N=7 control; N=6 hChR2	control, mean=33.8, SD=8.77; hChR2, mean=14.3, SD=14.4	two-tailed unpaired t-test	P=0.0119; t=3.009
3C	N=7 control; N=6 hChR2	control, OFF: mean=28.9; SD=15.754; control, ON: mean=25.8; SD=15.864; hChR2, OFF: mean=32.299; SD=9.429; hChR2, ON: mean=12.184; SD=9.898	two way repeated measures ANOVA; planned comparison between light on and light off for each group; Sidak's multiple comparisons	P=0.0148; SS=467.7; DF=1; MS=467.7; F(1,11)=8.336; adjusted P values for multiple comparisons: control, P=0.703; hChR2, P=0.0014
3D	N=7 control; N=6 hChR2		two way repeated measures ANOVA for each group	control: P>0.999; SS=21.27; DF=799; MS=0.0266; F(799, 9588)=0.0776; hChR2: P<0.0001; SS=540.1; DF=799; MS=0.676; F(799, 7990)=4.28
3E	N=7 control; N=6 hChR2	control, OFF: mean=6.239; SD=2.606; control, ON: mean=5.993; SD=2.630; hChR2, OFF: mean=6.578; SD=1.135; hChR2, ON: mean=3.404; SD=1.424	two way repeated measures ANOVA; planned comparison between light on and light off for each group; Sidak's multiple comparisons	P=0.0026; SS=13.85, DF=1; MS=13.85; F(1, 11)=14.97; adjusted P values for multiple comparisons: control, P=0.871; hChR2,P=0.0003
3F	N=7 control; N=6 hChR2	control, OFF: mean=1359.721; SD=1295.942; control, ON: mean=1300.71; SD=1185.064; hChR2, OFF: mean=1365.124; SD=563.159; hChR2, ON: mean=423.689; SD=432.615	two way repeated measures ANOVA; planned comparison between light on and light off for each group; Sidak's multiple comparisons	P=0.0139; SS=1257857, DF=1; MS=1257857; F(1, 11)=8.545; adjusted P values for multiple comparisons: control, P=0.951; hChR2,P=0.0027

3H	N=5 control; N=6 hChR2	control, mean=37.73, SD=12.51; hChR2, mean=21.94, SD=14.93	two-tailed unpaired t-test	P=0.0933; t=1.876
3I	N=5 control; N=6 hChR2	control, OFF: mean=18.025; SD=14.025; control, ON: mean=19.182; SD=11.329; hChR2, OFF: mean=15.051; SD=7.328; hChR2, ON: mean=8.841; SD=9.519	two way repeated measures ANOVA; planned comparison between light on and light off for each group; Sidak's multiple comparisons	P=0.133; SS=63.19; DF=1; MS=63.19; F(1,9)=2.724; adjusted P values for multiple comparisons: control, P=0.977; hChR2, P=0.102
3J	N=5 control; N=6 hChR2		two way repeated measures ANOVA for each group	control: P>0.999; SS=108.7; DF=799; MS=0.136; F(799, 6392)=0.686; hChR2: P<0.0001; SS=225.9; DF=799; MS=0.283; F(799, 7990)=2.356
3K	N=5 control; N=6 hChR2	control, OFF: mean=5.718; SD=2.293; control, ON: mean=5.778; SD=2.155; hChR2, OFF: mean=4.823; SD=1.288; hChR2, ON: mean=3.922; SD=1.32	two way repeated measures ANOVA; planned comparison between light on and light off for each group; Sidak's multiple comparisons	P=0.0642; SS=1.26; DF=1; MS=1.26; F(1,9)=4.448; adjusted P values for multiple comparisons: control, P=0.981; hChR2, P=0.0331
3L	N=5 control; N=6 hChR2	control, OFF: mean=1059.868; SD=948.379; control, ON: mean=1214.159; SD=868.175; hChR2, OFF: mean=798.553; SD=575.544; hChR2, ON: mean=530.696; SD=472.81	two way repeated measures ANOVA; planned comparison between light on and light off for each group; Sidak's multiple comparisons	P=0.0837; SS=243012; DF=1; MS=243012; F(1,9)=3.78; adjusted P values for multiple comparisons: control, P=0.592; hChR2, P=0.191
4B	N=6 control; N=6 hChR2		two way repeated measures ANOVA for each group	control: P=0.862; SS=126.4; DF=799; MS=0.158; F(799, 7990)=0.943; hChR2: P=0.0003; SS=344.7; DF=799; MS=0.431; F(799, 7990)=1.190
4C	N=6 control; N=6 hChR2	control, OFF: mean=7.001; SD=1.843; control, ON: mean=6.657; SD=1.424; hChR2, OFF: mean=6.301; SD=2.357; hChR2, ON: mean=5.187; SD=2.078	two way repeated measures ANOVA; planned comparison between light on and light off for each group; Sidak's multiple comparisons	P=0.2766; SS=0.887; DF=1; MS=0.887; F(1,10)=1.324; adjusted P values for multiple comparisons: control, P=0.733; hChR2, P=0.0789
4D	N=6 control; N=6 hChR2	control, OFF: mean=1910.372; SD=874.505; control, ON: mean=1633.975; SD=692.672; hChR2, OFF: mean=1497.517; SD=1202.637; hChR2, ON: mean=799.206; SD=807.669	two way repeated measures ANOVA; planned comparison between light on and light off for each group; Sidak's multiple comparisons	P=0.2674; SS=267017; DF=1; MS=267017; F(1,10)=1.38; adjusted P values for multiple comparisons: control, P=0.513; hChR2, P=0.0406
4F	N=7 control; N=7 hChR2		two way repeated measures ANOVA for each group	control: P>0.999; SS=1401; DF=39; MS=35.93; F(39, 468)=0.0277; hChR2: P=0.996; SS=27784; DF=39; MS=712.4; F(39, 546)=0.497
4G	N=7 control; N=7 hChR2	control, OFF: mean=6.217; SD=0.598; control, ON: mean=5.982; SD=0.685; hChR2, OFF: mean=6.345; SD=0.562; hChR2, ON: mean=6.534; SD=1.059	two way repeated measures ANOVA; planned comparison between light on and light off for each group; Sidak's multiple comparisons	P=0.324; SS=0.336; DF=1; MS=0.336; F(1,13)=1.051; adjusted P values for multiple comparisons: control, P=0.698; hChR2, P=0.765
4H	N=7 control; N=7 hChR2	control, OFF: mean=1.548; SD=0.329; control, ON: mean=1.472; SD=0.402; hChR2, OFF: mean=1.611; SD=0.204; hChR2, ON: mean=1.641; SD=0.408	two way repeated measures ANOVA; planned comparison between light on and light off for each group; Sidak's multiple comparisons	P=0.546; SS=0.021; DF=1; MS=0.021; F(1,13)=0.385; adjusted P values for multiple comparisons: control, P=0.8; hChR2, P=0.961
5H	N=2, n=22, untrained; N=2, n=24, trained	inhibitory, untrained: mean=79.431; SD=129.675; inhibitory, trained: mean=272.822; SD=238.078; excitatory, untrained: mean=38.464; SD=52.596; excitatory, trained: mean=48.566; SD=0.44.660	two way repeated measures ANOVA; Sidak's multiple comparisons	P=0.0026; SS(Type II)=192805; DF=1; MS=192805; F(1, 88)=9.636; adjusted P values for multiple comparisons: untrained excitatory vs. untrained inhibitory: P=0.917; untrained excitatory vs trained excitatory: P>0.999; untrained excitatory vs. trained inhibitory: P<0.0001; untrained inhibitory vs. trained excitatory: P=0.976; untrained inhibitory vs. trained inhibitory: P<0.0001; trained excitatory vs. trained inhibitory: P<0.0001
5I	N=2, n=22, untrained; N=2, n=24, trained	untrained: mean=5.371; SD=3.35; trained: mean=2.91; SD=3.122	two-tailed unpaired t-test	P=0.0136; t=2.571

S3D	N=4	layer V, lateral: mean=2248; SD=1463.277; medial: mean=4293.25; SD=2436.037; ventral: mean=7379.25; SD=3668.223; layer VI, lateral: mean=10029; SD=2314.81; medial: mean=13150.75; SD=3625.901; ventral: mean=19393.5; SD=4730.042	one way repeated measures ANOVA; Tukey's multiple comparisons for each layer independently	layer V: P=0.018; SS=53381560; DF=2; MS=26690780; F(1.763, 5.290)=9.747; adjusted P values for mutiple comparisons, lateral vs. ventral, P=0.0513; lateral vs. medial, P=0.22; ventral vs. medial, P=0.188; layer VI: P=0.0156; SS=181881481; DF=2; MS=90940741; F(1.847, 5.542)=9.867; adjusted P values for mutiple comparisons, lateral vs. ventral, P=0.0516; lateral vs. medial, P=0.333; ventral vs. medial, P=0.144
S3F	N=4	layer V anterior, lateral: mean=253.35; SD=237.666; ventral: mean=2778; SD=848.012; medial: mean=3019.5; SD=1836.014; layer V posterior, lateral: mean=1272.25; SD=1025.193; ventral: mean=2827.75; SD=2261.514; medial: mean=482.5; SD=288.437; layer VI anterior, lateral: mean=1462; SD=1149.387; ventral: mean=8351.5; SD=2076.224; medial: mean=9011.75; SD=1998.932; layer VI posterior, lateral: mean=5636; SD=684.726; ventral: mean=6887.75; SD=1827.258; medial: mean=2270.5; SD=993.550	two way ordinary ANOVA; Tukey's multiple comparisons	adjusted P values for mutiple comparisons, layer V anterior: lateral vs. ventral, P<0.0001; lateral vs. medial, P<0.0001; ventral vs. medial, P=0.821; layer V posterior: lateral vs. ventral, P=0.502; lateral vs. medial, P=0.017; ventral vs. medial, P=0.0014; layer VI anterior: lateral vs. ventral, P=0.0362; lateral vs. medial, P=0.0212; ventral vs. medial, P=0.964; layer VI posterior: lateral vs. ventral, P=0.243; lateral vs. medial, P=0.679; ventral vs. medial, P=0.0533;
S5A	N=4; n=740	2 to 6: mean=2139; SD=147.8; 6 to 10: mean=2168, SD=140; 10 to 14: mean=2171; SD=145.4; 14 and up: mean=2175; SD=145.1	two way repeated measures ANOVA; Tukey's multiple comparisons	P<0.0001; SS=613778; DF=3; MS=204593; F(3, 2956)=9.783; adjusted P values for multiple comparisons: 2 to 6 vs 6 to 10, P=0.0004; 2 to 6 vs. 10 to 14, P=0.0001; 2 to6 vs. 14 and up, P<0.0001; 6 to 10 vs. 10 to 14, P=0.996; 6 to 10 vs. 14 and up, P=0.832; 10 to 14 vss 14 and up, P=0.924
S5B	N=4; n=740	2 to 6: mean=-0.0504; SD=0.0344; 6 to 10: mean=-0.0552, SD=0.0359; 10 to 14: mean=-0.06; SD=0.0384; 14 and up: mean=-0.067; SD=0.0404	two way repeated measures ANOVA; Tukey's multiple comparisons	P<0.0001; SS=0.1049; DF=3; MS=0.035; F(3, 2956)=25.1; adjusted P values for multiple comparisons: 2 to 6 vs 6 to 10, P=0.0711; 2 to 6 vs. 10 to 14, P<0.0001; 2 to 6 vs. 14 and up, P<0.0001; 6 to 10 vs. 10 to 14, P=0.0657; 6 to 10 vs. 14 and up, P<0.0001; 10 to 14 vs 14 and up, P=0.0039
S6C	trial off: N=4; trial on: N=4	trial off: mean=26.352, SD=8.247; trial on: mean=7.973, SD=3.836	two-tailed unpaired t-test	P=0.0068; t=4.041
S6D	N=4	early: mean=0.196, SD=0.0946; late: mean=0.223, SD=0.111	two-tailed unpaired t-test	P<0.001; t=5.66
S7B	N=4; 2 to 6 early, n=8910; 2 to 6 late, n=11150; 6 to 10 early, n=2451; 6 to 10 late, n=3555; 10 to 12 early, n=1235; 10 to 14 late, n=2161; 14 and up early, n=1240; 14 and up late, n=2168	2 to 6 early: mean=3.608, SD=1.136; 2 to 6 late: mean=3.583, SD=1.153; 6 to 10 early: mean=7.68, SD=1.138; 6 to 10 late: mean=7.699, SD=1.155; 10 to 14 early: mean=11.82, SD=1.154; 10 to 14 late: mean=11.84, SD=1.17; 14 and up early: mean=20.16, SD=5.172; 14 and up late: mean=20.76, SD=5.634	ordinary one way ANOVA; Tukey's multiple comparison test for all comparisons	P<0.0001; SS=939946; DF=7; MS=134278; F(7,32862)=31349; adjusted P values for multiple comparisons: P=<0.0001 for all comparisons except the following; 2 to 6 early vs. 2 to 6 late: P=0.989; 6 to 10 early vs. 6 to 10 late: P>0.999; 10 to 14 early vs 10 to 14 late: P>0.999;
S8B	N=7 control; N=6 hChR2		two way repeated measures ANOVA for each group	control: P>0.999; SS=118; DF=799; MS=0.148; F(799, 9588)=0.0982; hChR2: P<0.001; SS=4335; DF=799; MS=5.426; F(799, 7191)=2.99
S8C	N=7 control; N=6 hChR2	control, OFF: mean=10.356; SD=2.502; control, ON: mean=10.181; SD=2.972; hChR2, OFF: mean=11.299; SD=1.107; hChR2, ON: mean=9.477; SD=1.468	two way repeated measures ANOVA; planned comparison between light on and light off for each group; Sidak's multiple comparisons	P=0.0327; SS=3.951, DF=1; MS=3.951; F(1, 10)=6.14; adjusted P values for multiple comparisons: control, P=0.905; hChR2,P=0.0098

S8D	N=7 control; N=6 hChR2	control, OFF: mean=2743.694; SD=1696.027; control, ON: mean=2592.003; SD=1790.459; hChR2, OFF: mean=3174.612; SD=406.003; hChR2, ON: mean=1931.767; SD=1132.402	two way repeated measures ANOVA; planned comparison between light on and light off for each group; Sidak's multiple comparisons	P=0.0451; SS=1736319, DF=1; MS=1736319; F(1, 10)=5.241; adjusted P values for multiple comparisons: control, P=0.865; hChR2,P=0.0132
S8E	N=7 control; N=6 hChR2		two way repeated measures ANOVA for each group	control: P>0.999; SS=24.19; DF=799; MS=0.0303; F(799, 9588)=0.0146; hChR2: P<0.001; SS=266.7; DF=799; MS=0.334; F(799, 7990)=3.301
S8F	N=7 control; N=6 hChR2	control, OFF: mean=4.198; SD=1; control, ON: mean=4.199; SD=1.013; hChR2, OFF: mean=4.411; SD=0.525; hChR2, ON: mean=2.635; SD=0.846	two way repeated measures ANOVA; planned comparison between light on and light off for each group; Sidak's multiple comparisons	P=0.004; SS=5.105, DF=1; MS=5.105; F(1, 11)=12.78; adjusted P values for multiple comparisons: control, P>0.999; hChR2, P=0.001
S8G	N=7 control; N=6 hChR2	control, OFF: mean=516.414; SD=318.142; control, ON: mean=592.114; SD=334.414; hChR2, OFF: mean=582.893; SD=292.127; hChR2, ON: mean=196.686; SD=193.102	two way repeated measures ANOVA; planned comparison between light on and light off for each group; Sidak's multiple comparisons	P=0.0149; SS=344654, DF=1; MS=344654; F(1, 11)=8.303; adjusted P values for multiple comparisons: control, P=0.751; hChR2, P=0.0145
S8H	N=7 control; N=6 hChR2	control, OFF: mean=47.215; SD=13.313; control, ON: mean=39.973; SD=12.779; hChR2, OFF: mean=51.820; SD=13.728; hChR2, ON: mean=36.125; SD=17.423	two way repeated measures ANOVA; planned comparison between light on and light off for each group; Sidak's multiple comparisons	P=0.186; SS=115.4; DF=1; MS=115.4; F(1, 11)=1.99; adjusted P values for multiple comparisons: control, P=0.195; hChR2, P=0.0088
S9B	N=4 control; N=6 hChR2	control, mean=34.868, SD=4.616; hChR2, mean=28.877, SD=2.984	two-tailed unpaired t-test	P=0.0357; t=2.521
S9C	N=7 control; N=6 hChR2	control, OFF: mean=33.545; SD=21.723; control, ON: mean=29.894; SD=21.536; hChR2, OFF: mean=34.392; SD=9.398; hChR2, ON: mean=26.736; SD=5.261	two way repeated measures ANOVA; planned comparison between light on and light off for each group; Sidak's multiple comparisons	P=0.389; SS=19.25; DF=1; MS=19.25; F(1, 8)=0.831; adjusted P values for multiple comparisons: control, P=0.53; hChR2, P=0.049
S9D	N=7 control; N=6 hChR2		two way repeated measures ANOVA for each group	control: P>0.999; SS=67.55; DF=799; MS=0.0846; F(799, 4794)=0.341; hChR2: P<0.001; SS=290.8; DF=799; MS=0.364; F(799, 7990)=1.274
S9E	N=7 control; N=6 hChR2	control, OFF: mean=4.713; SD=1.333; control, ON: mean=4.236; SD=1.375; hChR2, OFF: mean=5.729; SD=1.174; hChR2, ON: mean=4.679; SD=0.416	two way repeated measures ANOVA; planned comparison between light on and light off for each group; Sidak's multiple comparisons	P=0.36; SS=0.395, DF=1; MS=0.395; F(1, 8)=0.941; adjusted P values for multiple comparisons: control, P=0.549; hChR2, P=0.0452
S9F	N=7 control; N=6 hChR2	control, OFF: mean=530.737; SD=234.22; control, ON: mean=478.182; SD=206.218; hChR2, OFF: mean=734.782; SD=289.356; hChR2, ON: mean=548.276; SD=101.972	two way repeated measures ANOVA; planned comparison between light on and light off for each group; Sidak's multiple comparisons	P=0.36; SS=24488, DF=1; MS=24488; F(1, 8)=1.122; adjusted P values for multiple comparisons: control, P=0.862; hChR2, P=0.0997
S10A	N=5 control; N=5 hChR2		two way repeated measures ANOVA for each group	control: P>0.999; SS=1815; DF=799; MS=2.272; F(799, 6392)=0.723; hChR2: P>0.999; SS=1619; DF=799; MS=2.027; F(799, 6392)=0.729
S10B	N=5 control; N=5 hChR2	control, OFF: mean=9.939; SD=2.136; control, ON: mean=9.992; SD=1.914; hChR2, OFF: mean=8.874; SD=0.899; hChR2, ON: mean=9.532; SD=1.67	two way repeated measures ANOVA; planned comparison between light on and light off for each group; Sidak's multiple comparisons	P=0.52; SS=0.457, DF=1; MS=0.457; F(1, 8)=0.454; adjusted P values for multiple comparisons: control, P=0.996; hChR2, P=0.552
S10C	N=5 control; N=5 hChR2	control, OFF: mean=2272.762; SD=1815.832; control, ON: mean=2018.416; SD=3239.149; hChR2, OFF: mean=2219.905; SD=902.005; hChR2, ON: mean=2010.021; SD=854.026	two way repeated measures ANOVA; planned comparison between light on and light off for each group; Sidak's multiple comparisons	P=0.96; SS=2471, DF=1; MS=2471; F(1, 8)=0.00264; adjusted P values for multiple comparisons: control, P=0.903; hChR2, P=0.933

S10D	N=5 control; N=6 hChR2		two way repeated measures ANOVA for each group	control: P>0.999; SS=35.75; DF=799; MS=0.0447; F(799, 6392)=0.265; hChR2: P<0.001; SS=192.5; DF=799; MS=0.241; F(799, 7990)=2.583
S10E	N=5 control; N=6 hChR2	control, OFF: mean=4.657; SD=1.529; control, ON: mean=4.793; SD=1.524; hChR2, OFF: mean=4.156; SD=1.157; hChR2, ON: mean=3.471; SD=0.906	two way repeated measures ANOVA; planned comparison between light on and light off for each group; Sidak's multiple comparisons	P=0.0405; SS=0.92, DF=1; MS=0.92; F(1, 9)=5.718; adjusted P values for multiple comparisons: control, P=0.844; hChR2, P=0.0316
S10F	N=5 control; N=6 hChR2	control, OFF: mean=624.243; SD=651.045; control, ON: mean=760.872; SD=550.083; hChR2, OFF: mean=506.562; SD=452.633; hChR2, ON: mean=384.972; SD=345.679	two way repeated measures ANOVA; planned comparison between light on and light off for each group; Sidak's multiple comparisons	P=0.092; SS=90922, DF=1; MS=90922; F(1, 9)=0.092; adjusted P values for multiple comparisons: control, P=0.376; hChR2, P=0.393
S11A	N=6 control; N=6 hChR2		two way repeated measures ANOVA for each group	control: P=0.862; SS=126.4; DF=799; MS=0.158; F(799, 7990)=0.943; hChR2: P=0.0003; SS=344.7; DF=799; MS=0.431; F(799, 7990)=1.190
S11B	N=6 control; N=6 hChR2	control, mean=32.12, SD=7.24; hChR2, mean=24.47, SD=10.2	two-tailed unpaired t-test	P=0.164; t=1.5
S11C	N=6 control; N=6 hChR2	control, OFF: mean=35.745; SD=14.184; control, ON: mean=33.729; SD=14.296; hChR2, OFF: mean=39.483; SD=18.945; hChR2, ON: mean=32.078; SD=17.742	two way repeated measures ANOVA; planned comparison between light on and light off for each group; Sidak's multiple comparisons	P=0.092; SS=90922, DF=1; MS=90922; F(1, 9)=0.092; adjusted P values for multiple comparisons: control, P=0.376; hChR2, P=0.393
S11D	N=6 control; N=6 hChR2		two way repeated measures ANOVA for each group	control: P=0.999; SS=455.2; DF=799; MS=0.57; F(799, 7990)=0.852; hChR2: P<0.0001; SS=2210; DF=799; MS=2.766; F(799, 7990)=3.023
S11E	N=6 control; N=6 hChR2	control, OFF: mean=10.226; SD=2.09; control, ON: mean=9.833; SD=2.042; hChR2, OFF: mean=9.7; SD=1.856; hChR2, ON: mean=9.085; SD=2.305	two way repeated measures ANOVA; planned comparison between light on and light off for each group; Sidak's multiple comparisons	P=0.815; SS=0.0742, DF=1; MS=0.0742; F(1, 10)=0.0578; adjusted P values for multiple comparisons: control, P=0.808; hChR2, P=0.602
S11F	N=6 control; N=6 hChR2	control, OFF: mean=3141.464; SD=1251.3; control, ON: mean=2359.667; SD=1089.946; hChR2, OFF: mean=2577.152; SD=1481.397; hChR2, ON: mean=1990.271; SD=1597.535	two way repeated measures ANOVA; planned comparison between light on and light off for each group; Sidak's multiple comparisons	P=0.872; SS=16564, DF=1; MS=16564; F(1, 10)=0.0273; adjusted P values for multiple comparisons: control, P=0.523; hChR2, P=0.394
S11G	N=6 control; N=6 hChR2		two way repeated measures ANOVA for each group	control: P>0.999; SS=87.59; DF=799; MS=0.11; F(799, 7990)=0.683; hChR2: P<0.0001; SS=266.7; DF=799; MS=0.334; F(799, 7990)=3.301
S11H	N=6 control; N=6 hChR2	control, OFF: mean=5.111; SD=1.303; control, ON: mean=4.669; SD=0.314; hChR2, OFF: mean=4.1; SD=1.297; hChR2, ON: mean=3.263; SD=1.118	two way repeated measures ANOVA; planned comparison between light on and light off for each group; Sidak's multiple comparisons	P=0.667; SS=0.231, DF=1; MS=0.231; F(1, 10)=0.197; adjusted P values for multiple comparisons: control, P=0.746; hChR2, P=0.379
S11I	N=6 control; N=6 hChR2	control, OFF: mean=1126.27; SD=492.354; control, ON: mean=939.424; SD=245.927; hChR2, OFF: mean=663.804; SD=560.802; hChR2, ON: mean=332.098; SD=352.319	two way repeated measures ANOVA; planned comparison between light on and light off for each group; Sidak's multiple comparisons	P=0.614; SS=31477, DF=1; MS=31477; F(1, 10)=0.271; adjusted P values for multiple comparisons: control, P=0.597; hChR2, P=0.231
S11K	N=10 control; N=11 hChR2		two way repeated measures ANOVA for each group	control: P>0.999; SS=244.8; DF=799; MS=0.306; F(799, 15980)=0.654; hChR2: P<0.0001; SS=336.2; DF=799; MS=0.421; F(799, 15980)=1.347
S11L	N=10 control; N=11 hChR2	control, OFF: mean=7.439; SD=1.521; control, ON: mean=7.812; SD=1.763; hChR2, OFF: mean=8.011; SD=1.636; hChR2, ON: mean=8.814; SD=1.547	two way repeated measures ANOVA; planned comparison between light on and light off for each group; Sidak's multiple comparisons	P=0.151; SS=0.507, DF=1; MS=0.501; F(1, 20)=2.232; adjusted P values for multiple comparisons: control, P=0.156; hChR2, P=0.0016

S11M	N=10 control; N=11 hChR2	control, OFF: mean=1210.406; SD=507.691; control, ON: mean=1353.36; SD=563.168; hChR2, OFF: mean=1366.513; SD=574.794; hChR2, ON: mean=1628.167; SD=562.261	two way repeated measures ANOVA; planned comparison between light on and light off for each group; Sidak's multiple comparisons	P=0.202; SS=38747, DF=1; MS=38747; F(1, 20)=1.742; adjusted P values for multiple comparisons: control, P=0.0708; hChR2, P=0.0011
S12C	N=7 control; N=7 hChR2		two way repeated measures ANOVA for each group	control: P>0.999; SS=1401; DF=39; MS=35.93; F(39, 468)=0.0277; hChR2: P=0.996; SS=27784; DF=39; MS=712.4; F(39, 546)=0.497
S12D	N=6 control; N=6 hChR2	control, OFF: mean=10517.172; SD=1456.715; control, ON: mean=10486.401; SD=1789.038; hChR2, OFF: mean=9777.595; SD=726.394; hChR2, ON: mean=9633.715; SD=1299.517	two way repeated measures ANOVA; planned comparison between light on and light off for each group; Sidak's multiple comparisons	P=0.776; SS=19191, DF=1; MS=19191; F(1, 10)=0.0855; adjusted P values for multiple comparisons: control, P=0.992; hChR2, P=0.848

Supplementary Table 3. Statistics summary

Summary of all statistics by figure panel.
