# Supplementary material for: Corticothalamic neurons in motor cortex have a permissive role in motor execution
Source: Nat Commun. 2025 May 21;16:4735. doi: 10.1038/s41467-025-59954-1 (PMC12095750; doi:10.1038/s41467-025-59954-1)
Supplement: Supplementary file 2 — Description Of Additional Supplementary File [file 41467_2025_59954_MOESM2_ESM.pdf]

Description of Additional supplementary file

Supplementary Movie 1. Representative example of light off trial executed by a control mouse. Trial start and reward are denoted. LED flashes also denote reward delivery.

Supplementary Movie 2. Representative example of light on trial executed by a control mouse. Trial start and concurrent opsin activating light are denoted.

Supplementary Movie 3. Representative example of light off trial executed by an hChR2 mouse. Trial start and reward are denoted. LED flashes also denote reward delivery.

Supplementary Movie 4. Representative example of light on trial executed by an hChR2 mouse. Trial start and concurrent opsin activating light are denoted.
