# Supplementary material for: Corticothalamic neurons in motor cortex have a permissive role in motor execution
Source: Nat Commun. 2025 May 21;16:4735. doi: 10.1038/s41467-025-59954-1 (PMC12095750; doi:10.1038/s41467-025-59954-1)
Supplement: Supplementary file 7 — Reporting Summary [file 41467_2025_59954_MOESM7_ESM.pdf]

nature portfolio

Corresponding author(s): Rui Costa

Last updated by author(s): 4/16/2025

Reporting Summary

Nature Portfolio wishes to improve the reproducibility of the work that we publish. This form provides structure for consistency and transparency in reporting. For further information on Nature Portfolio policies, see our Editorial Policies and the Editorial Policy Checklist.

Statistics

For all statistical analyses, confirm that the following items are present in the figure legend, table legend, main text, or Methods section.

n/a	Confirmed	
☐	☒	The exact sample size (*n*) for each experimental group/condition, given as a discrete number and unit of measurement
☐	☒	A statement on whether measurements were taken from distinct samples or whether the same sample was measured repeatedly
☐	☒	The statistical test(s) used AND whether they are one- or two-sided<br>*Only common tests should be described solely by name; describe more complex techniques in the Methods section.*
☒	☐	A description of all covariates tested
☐	☒	A description of any assumptions or corrections, such as tests of normality and adjustment for multiple comparisons
☐	☒	A full description of the statistical parameters including central tendency (e.g. means) or other basic estimates (e.g. regression coefficient) AND variation (e.g. standard deviation) or associated estimates of uncertainty (e.g. confidence intervals)
☐	☒	For null hypothesis testing, the test statistic (e.g. *F*, *t*, *r*) with confidence intervals, effect sizes, degrees of freedom and *P* value noted<br>*Give P values as exact values whenever suitable.*
☒	☐	For Bayesian analysis, information on the choice of priors and Markov chain Monte Carlo settings
☒	☐	For hierarchical and complex designs, identification of the appropriate level for tests and full reporting of outcomes
☒	☐	Estimates of effect sizes (e.g. Cohen's *d*, Pearson's *r*), indicating how they were calculated

Our web collection on statistics for biologists contains articles on many of the points above.

Software and code

Policy information about availability of computer code

Data collection	Behavioral data was collected with MATLAB 2015. Calcium Imaging data was collected with Prarie View
Data analysis	Behavioral data was analyzed with MATLAB 2021b using standard functions. Prism 9 was used to generate plots and for statistical analysis. ClearMap (Python) was used for analysis of AdipoClear projection mapping. BrainJ (FIJI) was used for anatomical mapping. Single-cell RNA sequencing data was analyzed using Cellranger (10x Genomics) and Seurat v4.0.0.

For manuscripts utilizing custom algorithms or software that are central to the research but not yet described in published literature, software must be made available to editors and reviewers. We strongly encourage code deposition in a community repository (e.g. GitHub). See the Nature Portfolio guidelines for submitting code & software for further information.

Data

Policy information about availability of data

All manuscripts must include a data availability statement. This statement should provide the following information, where applicable:

- Accession codes, unique identifiers, or web links for publicly available datasets
- A description of any restrictions on data availability
- For clinical datasets or third party data, please ensure that the statement adheres to our policy

Single-cell RNA-sequencing data had been deposited in the Gene Expression Omnibus under the accession number GSE292904 . Source data are provided as Source

data files. Larger data sets have been uploaded to Figshare under the following DOI: 10.6084/m9.figshare.28111892 . Any other data supporting this study is available upon request.

Research involving human participants, their data, or biological material

Policy information about studies with human participants or human data. See also policy information about sex, gender (identity/presentation), and sexual orientation and race, ethnicity and racism.

Reporting on sex and gender	*Use the terms sex (biological attribute) and gender (shaped by social and cultural circumstances) carefully in order to avoid confusing both terms. Indicate if findings apply to only one sex or gender; describe whether sex and gender were considered in study design; whether sex and/or gender was determined based on self-reporting or assigned and methods used. Provide in the source data disaggregated sex and gender data, where this information has been collected, and if consent has been obtained for sharing of individual-level data; provide overall numbers in this Reporting Summary. Please state if this information has not been collected. Report sex- and gender-based analyses where performed, justify reasons for lack of sex- and gender-based analysis.*
Reporting on race, ethnicity, or other socially relevant groupings	*Please specify the socially constructed or socially relevant categorization variable(s) used in your manuscript and explain why they were used. Please note that such variables should not be used as proxies for other socially constructed/relevant variables (for example, race or ethnicity should not be used as a proxy for socioeconomic status). Provide clear definitions of the relevant terms used, how they were provided (by the participants/respondents, the researchers, or third parties), and the method(s) used to classify people into the different categories (e.g. self-report, census or administrative data, social media data, etc.) Please provide details about how you controlled for confounding variables in your analyses.*
Population characteristics	*Describe the covariate-relevant population characteristics of the human research participants (e.g. age, genotypic information, past and current diagnosis and treatment categories). If you filled out the behavioural & social sciences study design questions and have nothing to add here, write "See above."*
Recruitment	*Describe how participants were recruited. Outline any potential self-selection bias or other biases that may be present and how these are likely to impact results.*
Ethics oversight	*Identify the organization(s) that approved the study protocol.*

Note that full information on the approval of the study protocol must also be provided in the manuscript.

Field-specific reporting

Please select the one below that is the best fit for your research. If you are not sure, read the appropriate sections before making your selection.

☒ Life sciences ☐ Behavioural & social sciences ☐ Ecological, evolutionary & environmental sciences

For a reference copy of the document with all sections, see nature.com/documents/nr-reporting-summary-flat.pdf

Life sciences study design

All studies must disclose on these points even when the disclosure is negative.

Sample size	Sample sizes were determined based on similar behavioral studies in the field (see references 23 and 27). No statistical methods were used to calculate sample size.
Data exclusions	Preestablished criteria was used for any exclusions. For studies reliant on stereotactic injections, animals were excluded if targeting was not as specific as intended.
Replication	All experiments contained animal replicates with numbers noted throughout the manuscript. Effect were examined in each animal before this was aggregated for cohort level analysis.
Randomization	Mice were randomly assigned to control or experimental groups.
Blinding	Behavioral analysis was performed blind to whether animals were in control or experimental groups until aggregated for final comparisons.

Reporting for specific materials, systems and methods

We require information from authors about some types of materials, experimental systems and methods used in many studies. Here, indicate whether each material, system or method listed is relevant to your study. If you are not sure if a list item applies to your research, read the appropriate section before selecting a response.

Materials & experimental systems

n/a	Involved in the study
☐	☒ Antibodies
☒	☐ Eukaryotic cell lines
☒	☐ Palaeontology and archaeology
☐	☒ Animals and other organisms
☒	☐ Clinical data
☒	☐ Dual use research of concern
☒	☐ Plants

Methods

n/a	Involved in the study
☒	☐ ChIP-seq
☐	☒ Flow cytometry
☒	☐ MRI-based neuroimaging

Antibodies

Antibodies used

chicken GFP antibody (Aves; GFP1020; polyclonal); dilution of 1:2500
rabbit RFP antibody (Rockland; 600-401-379; polyclonal); dilution of 1:2500
donkey anti-rabbit Alexa Fluor 647 (ThermoFisher Scientific; A31573; polyclonal); dilution of 1:2500
donkey anti-chicken Alexa Fluor 647 (ThermoFisher Scientific; A78952; polyclonal); dilution of 1:2500

Validation

chicken GFP Quality Control statement from Aves:
Antibodies were analyzed by western blot analysis (1:5000 dilution) and immunohistochemistry (1:500 dilution) using transgenic mice expressing the GFP gene product. Western blots were performed using BlokHen® (Aves Labs) as the blocking reagent, and HRP-labeled goat anti-chicken antibodies (Aves Labs, Cat. #H-1004) as the detection reagent. Immunohistochemistry used tetramethyl rhodamine-labeled anti-chicken IgY.

rabbit RFP Purity/Specificity statement from Rockland:
This product was prepared from monospecific antiserum by immunoaffinity chromatography using Red Fluorescent Protein (Discosoma) coupled to agarose beads followed by solid phase adsorption(s) to remove any unwanted reactivities. Expect reactivity against RFP and its variants: mCherry, tdTomato, mBanana, mOrange, mPlum, mOrange and mStrawberry. Assay by immunoelectrophoresis resulted in a single precipitin arc against anti-Rabbit Serum and purified and partially purified Red Fluorescent Protein (Discosoma). No reaction was observed against Human, Mouse or Rat serum proteins.

donkey anti-rabbit Alexa Fluor 647 statement from ThermoFisher Scientific:
To minimize cross-reactivity, these donkey anti-rabbit IgG whole antibodies have been affinity-purified and show a published cross-reactivity to rat IgG. Cross-adsorption or pre-adsorption is a purification step to increase specificity of the antibody resulting in higher sensitivity and less background staining. The secondary antibody solution is passed through a column matrix containing immobilized serum proteins from potentially cross-reactive species. Only the nonspecific-binding secondary antibodies are captured in the column, and the highly specific secondaries flow through.

donkey anti-chicken Alexa Fluor 647 statement from ThermoFisher Scientific:
This donkey anti-chicken IgY (H+L) whole secondary antibody has been affinity-purified and shows minimum cross-reactivity to bovine, goat, guinea pig, syrian hamster, horse, human, mouse, rabbit, rat and sheep serum proteins. Cross-adsorption or pre-adsorption is a purification step to increase specificity of the antibody resulting in higher sensitivity and less background staining. The secondary antibody solution is passed through a column matrix containing immobilized serum proteins from potentially cross-reactive species. Only the nonspecific-binding secondary antibodies are captured in the column, and the highly specific secondary antibodies flow through.

Animals and other research organisms

Policy information about studies involving animals; ARRIVE guidelines recommended for reporting animal research, and Sex and Gender in Research

Laboratory animals

The strains used were: C57BL6/J (Jackson Laboratories, 000664), FoxP2-Cre (Jackson Laboratories, 030541), and Ntsr1-Cre (Mutant Mouse Resource & Research Centers, 030648-UCD). Adult mice, aged 2–6 months, were used for all experiments

Wild animals

This study did not involve wild animals.

Reporting on sex

Adult mice of both sexes, aged 2–6 months, were used for all experiments, except for RNA sequencing which only used males aged 3 months.

Field-collected samples

This study did not involve samples field-collected samples.

Ethics oversight

All experiments and procedures were performed according to National Institutes of Health (NIH) guidelines and approved by the Institutional Animal Care and Use Committee of Columbia University.

Note that full information on the approval of the study protocol must also be provided in the manuscript.

Plants

Seed stocks	*Report on the source of all seed stocks or other plant material used. If applicable, state the seed stock centre and catalogue number. If plant specimens were collected from the field, describe the collection location, date and sampling procedures.*
Novel plant genotypes	*Describe the methods by which all novel plant genotypes were produced. This includes those generated by transgenic approaches, gene editing, chemical/radiation-based mutagenesis and hybridization. For transgenic lines, describe the transformation method, the number of independent lines analyzed and the generation upon which experiments were performed. For gene-edited lines, describe the editor used, the endogenous sequence targeted for editing, the targeting guide RNA sequence (if applicable) and how the editor was applied.*
Authentication	*Describe any authentication procedures for each seed stock used or novel genotype generated. Describe any experiments used to assess the effect of a mutation and, where applicable, how potential secondary effects (e.g. second site T-DNA insertions, mosiacism, off-target gene editing) were examined.*

Flow Cytometry

Plots

Confirm that:

☐ The axis labels state the marker and fluorochrome used (e.g. CD4-FITC).

☐ The axis scales are clearly visible. Include numbers along axes only for bottom left plot of group (a 'group' is an analysis of identical markers).

☐ All plots are contour plots with outliers or pseudocolor plots.

☐ A numerical value for number of cells or percentage (with statistics) is provided.

Methodology

Sample preparation	M1 was quickly dissected, and single-cell suspensions were generated using the previously described protocol optimized for preserving transcriptional state with the following modifications (Hrvatin, S. et al. Single-cell analysis of experience-dependent transcriptomic states in the mouse visual cortex. Nat Neurosci 21, 120-129 (2018). https://doi.org:10.1038/s41593-017-0029-5). The dissected M1 tissue was further triturated with spring scissors. Incubation with papain was conducted using a dialysis membrane (Fisher Scientific, Slide-a-Lyzer MINI Dialysis Device,10k molecular weight cut-off) suspended in a beaker of dissociation solution allowing for constant oxygenation (95% O2 and 5% CO2) of the solution without disrupting enzyme integrity within the dialysis membrane. After enzymatic dissociation, the tissue was broken into a single-cell suspension by gently pipetting using wide-bore pipette tips. The sample was cleaned using the Debris Removal Solution (Miltenyi Biotec) as described by the manufacturer. Cells were suspended in dissociation solution containing 0.04% BSA, Vybrant DyeCycle Ruby for exclusion debris, and RNAase inhibitors.
Instrument	Cell sorting was performed using a FACSAria Cell Sorter (Becton Dickinson) with a 130 um nozzle at 12 PSI using the following laser lines and filters: CaMPARI Green, 488 nm, 530/30 bandpass; CaMPARI Red, 561 nm, 610/20 bandpass; DyeCycle Ruby, 637 nm, 670/30 bandpass.
Software	Data was collected with FACSDiva software (Becton Dickinson).
Cell population abundance	Population abundance was not calculated.
Gating strategy	An initial gate using DyeCycle ruby was used to identify live cells. CaMPARI gates were set using a sample not expressing CaMPARI and a sample not exposed to photoconversion light. Any cells with detectable red fluorescence were collected.

☐ Tick this box to confirm that a figure exemplifying the gating strategy is provided in the Supplementary Information.
